# Genomic epidemiology reveals the origins and transmission dynamics of chikungunya virus in China

**DOI:** 10.1186/s40249-026-01465-2

**Published:** 2026-06-04

**Authors:** Wei Chang, Mengyuan Zheng, Yanxian Jiang, Gaowen Liu, Xiao Chen, Xue Wang, Yan Guo, Li Liu, Yue Feng, Xueshan Xia

**Affiliations:** 1 Present Address: Faculty of Life Science and Technology & Yunnan Provincial Key Laboratory of Public Health and Biosafety, Kunming University of Science and Technology, No.727 Jingming Road, Chenggong Campus, Kunming, Yunnan China; 2 Yunnan Provincial Key Laboratory of Public Health and Biosafety, Kunming Medical University, Kunming, Yunnan China

**Keywords:** Chikungunya virus, Genomic analysis, Phylogeographic reconstruction, China

## Abstract

**Background:**

The chikungunya virus (CHIKV) poses a significant public health threat in China, driven by travel-related introductions and local outbreaks. A comprehensive understanding of the spatiotemporal dynamics, viral genetic diversity, and origins of these outbreaks is imperative for surveillance and control measures. This study aimed to characterize the spatiotemporal dynamics of CHIKV in China and elucidate the evolutionary origins of outbreak lineages.

**Methods:**

A systematic collection of epidemiological data on CHIKV cases in China was conducted up to December 2025, resulting in the identification of 416 imported cases and 25,948 local cases. A comprehensive retrieval of all publicly available CHIKV genomes from China was conducted. The composition of viral lineages was determined by employing maximum likelihood phylogeny. Substitution rates were estimated, and time-scaled transmission clusters were generated using Bayesian evolutionary analysis. Discrete phylogeographic reconstruction was used to infer spatial diffusion pathways, with statistical support evaluated using Bayes Factors.

**Results:**

From 1987 to 2025, imported chikungunya cases in China mainly originated from Southeast Asia, especially Myanmar, and were accompanied by several major local outbreaks. Significant outbreaks were reported in Guangdong (2010 and 2025), Yunnan (2019), and Taiwan (2019), with the largest outbreak occurring in Guangdong in 2025 and involving 25,335 cases. Genomic analysis of 314 sequences revealed that the East/Central/South African lineage was the most prevalent, accounting for 92.0% of cases. This lineage includes the Indian Ocean (IOL) and Central African clades. The Asian lineage accounted for the remaining 8.0%. Bayesian analysis identified four independent outbreak clusters. Phylogeographic data supported the conclusion that there were multiple introductions. The IOL clusters spread from Africa and South Asia to Southeast Asia. The 2025 Guangdong outbreak cluster (Central African clade) suggests a possible association with introduction from Africa. The Asian lineage circulated persistently in Southeast Asia before entering Yunnan Province.

**Conclusions:**

CHIKV outbreaks in China are linked to imported cases, and outbreak lineages consistently match recent imports. The virus entered through various, geographically independent routes. These findings underscore the need for genomic-informed surveillance at key entry points and in high-risk regions to anticipate and prevent local epidemics.

**Graphical Abstract:**

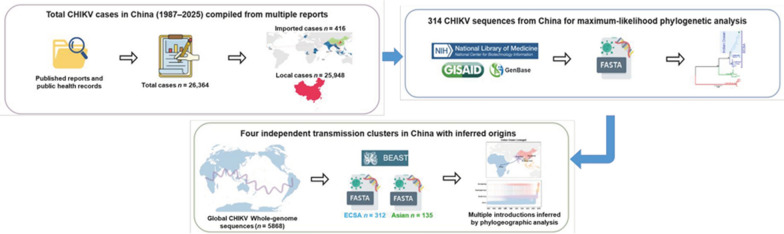

**Supplementary Information:**

The online version contains supplementary material available at 10.1186/s40249-026-01465-2.

## Background

Chikungunya virus (CHIKV) is an alphavirus transmitted primarily by *Aedes albopictus* and *Ae. aegypti* mosquitoes [1]. Clinical manifestations of CHIKV infection include fever, rash, and severe arthralgia. In certain cases, particularly among neonates and the elderly, more severe complications such as meningitis and acute liver injury have been observed [2, 3]. Since its initial identification in Tanzania in 1952, CHIKV has been responsible for numerous large-scale epidemics in tropical and subtropical regions worldwide [4]. In 2025, for instance, Brazil reported the highest number of cases in a single country, with over 90,000 confirmed infections and 111 fatalities [4]. Concurrently, Réunion, an island and overseas department of France located in the south-west Indian Ocean, documented 54,517 confirmed cases and 40 deaths. At the same time, China encountered its most substantial recorded CHIKV outbreak, with 25,335 confirmed cases [4, 5]. Currently, no specific antiviral drugs are available for treating CHIKV infection, and clinical management primarily consists of supportive care. Despite the approval of two vaccines for use in high-risk groups in several countries, their global availability remains constrained [6]. Consequently, chikungunya fever has emerged as a pressing global public health threat.

The genome of the CHIKV is estimated to range from 11.8 to 12 kb in length. Phylogenetic analyses categorize CHIKV into three primary lineages: West African, East/Central/South African (ECSA), and Asian [2]. The ECSA lineage is further divided into three distinct clades: the Indian Ocean clade (IOL), the Central African clade, and the East/South African clade [7]. The West African lineage, the first to be discovered, has remained geographically confined to the rainforest ecosystems of West Africa. The Asian lineage, which was introduced to Asia from Africa between the late nineteenth and mid-twentieth centuries, has caused multiple localized outbreaks across Asia. This lineage subsequently disseminated to the Americas in 2013, precipitating widespread outbreaks in the Caribbean and other regions of the Americas. The ECSA lineage, particularly the IOL clade, has demonstrated a noteworthy capacity for rapid global dispersal, as evidenced by numerous studies. The introduction of the IOL clade in the Kenyan coastal region in 2004 led to widespread epidemics from 2005 to 2007, exerting a substantial impact on the Indian Ocean region and parts of Asia [2]. Recent studies have indicated that the ECSA-Central African clade has exhibited notable activity, with outbreaks reported on Réunion Island, France, during 2024–2025, as well as a concurrent local epidemic in China [8, 9]. The ECSA lineage is of particular concern on an international scale due to its association with prolonged arthralgia [10].

In the context of frequent international travel, CHIKV can be introduced into new regions by infected travelers, potentially leading to local transmission in areas with competent vectors and suitable environmental conditions. Since the initial documented instance of importation in 2006, China has predominantly reported imported cases of CHIKV, with sporadic importations persisting [11, 12]. Furthermore, these importation events have repeatedly triggered local outbreaks in southern China, notably in the years 2010, 2019, and 2025 [13–15]. The most recent outbreak rapidly established effective local transmission chains, resulting in an unprecedented epidemic within weeks [16]. However, there is a paucity of systematic analysis that integrates the spatiotemporal dynamics of importation with the genomic factors driving local spread. Consequently, the identification of outbreak origins and the development of targeted measures based on forecasting transmission risk remain constrained.

Phylogenetic and spatiotemporal dynamic analyses employing viral genomic data have been demonstrated to trace the origins of outbreaks, reveal hidden transmission chains, and predict patterns of spread [17]. These analyses provide a scientific basis for accurate border screening and targeted local interventions. In this study, we integrated comprehensive epidemiological information with viral genomic sequences to systematically characterize the spatiotemporal dynamics of imported CHIKV cases in China. The objective of this study is to elucidate the evolutionary origins and salient characteristics of the viral lineages responsible for local outbreaks. The findings of this study offer essential evidence to support the implementation of early risk-warning systems and to enhance prevention and control strategies for chikungunya fever in China.

## Methods

### Data collection

A comprehensive investigation was conducted to gather data on reported cases of CHIKV infection in China. This endeavor entailed a meticulous search and compilation of pertinent information from published literature and outbreak reports. A comprehensive literature search was conducted in the PubMed and China National Knowledge Infrastructure (CNKI) databases, with a cutoff date of December 13, 2025. The search term employed in the PubMed database was "(chikungunya OR CHIKV) AND China," while corresponding combinations of Chinese keywords were utilized in CNKI. A curated selection of research articles and official outbreak reports was utilized to extract pertinent case information, including the reporting region, reporting date, and the number of cases (Fig. S1). The imported cases focused on individuals diagnosed with the infection after entering China from another country, with confirmed infection and a clear entry date into China. The definition of a confirmed case was in accordance with the criteria established by the World Health Organization, which required that a clinical specimen meet at least one of the following laboratory test results: Virus isolation; positive CHIKV nucleic acid test; CHIKV-specific IgM antibody detection confirmed by neutralization test; or seroconversion or ≥ fourfold increase in specific antibody titers between acute and convalescent-phase serum samples [4].

A comprehensive retrieval of all CHIKV genome sequences collected in China was conducted from three prominent public databases: the National Center for Biotechnology Information (NCBI) GenBank, the Global Initiative on Sharing All Influenza Data (GISAID), and the China National Genomics Data Center (GenBase). The download of data was completed by December 13, 2025. For subsequent analysis, we elected to retain exclusively those sequences for which the collection location and date were clearly documented.

### Phylogenetic analysis

A total of 618 CHIKV sequences with sampling locations in China were retrieved from three public databases. To ensure data quality and reduce redundancy, several filtering steps were applied. Duplicate sequences (*n* = 231), defined as sequences with > 99.9% nucleotide identity from the same sampling location and time, were removed. Additionally, sequences from non-human hosts (*n* = 56), laboratory-passaged strains, and sequences lacking clear sampling dates (*n* = 17) were excluded. After this curation process, a total of 314 sequences were retained for subsequent phylogenetic analyses (Fig. S1). Due to variations in the genomic regions covered by the obtained sequences, these were divided into six datasets based on their alignment coverage. For each dataset, reference sequences representing the known major CHIKV lineages (West African, ECSA, and Asian) from GenBank were included. Multiple sequence alignment was performed using MAFFT v7.310 (developed by Kazutaka Katoh and Daron Standley, Immunology Frontier Research Center, Osaka University, Osaka, Japan), and the alignments were subsequently trimmed to eliminate unreliable sites that contained excessive gaps. Maximum likelihood phylogenetic trees were constructed using IQ-TREE v2.4.0 (developed by the Center for Integrative Bioinformatics, Vienna, Austria). Optimal nucleotide substitution models were determined using ModelFinder; GTR + F + I + G4 was selected for all datasets. The support for the phylogenetic tree nodes was assessed using 1000 bootstrap replicates. Trees were visualized and annotated using FigTree v1.4.4 (developed by Andrew Rambaut, Institute of Evolutionary Biology, University of Edinburgh, UK).

### Phylogeographic analyses

A Bayesian discrete phylogeographic analysis was conducted to trace the geographic origins and spread of CHIKV outbreaks in China. Publicly available CHIKV genome sequences, with a minimum coverage of 60%, were retrieved from prominent databases including NCBI, GISAID, and GenBase. Duplicate sequences were removed using SeqKit v2.3.0 (developed by Wei Shen, Third Military Medical University, Chongqing, China), and sequences lacking collection date or precise geographic information were excluded, resulting in a dataset of 5868 sequences. Sampling locations were categorized into discrete geographic regions, including North America, South America, Europe, Africa, Oceania, South Asia, West Asia, and Southeast Asia. To focus on viral spread within China, Yunnan and Guangdong provinces were defined as separate geographic states. A stratified subsampling strategy was employed to balance computational feasibility with the preservation of genetic and geographic diversity [18]. Within each region, transmission clusters were identified using ClusterPicker v1.2.3 (developed by Samantha Lycett et al., University of Edinburgh, UK) based on phylogenetic tree topology, branch support, and genetic distance. Branch support was assessed using the approximate likelihood ratio test, with both initial and main support thresholds set to 0.9. Genetic distance was defined as the maximum pairwise distance within clusters, and clusters were retained only if the maximum intra-cluster distance was ≤ 4.5%. To reduce redundancy while preserving genetic diversity, representative sequences were selected from each cluster, with 3–10 sequences retained per cluster depending on cluster size. For sequences collected in Brazil within the South American dataset (*n* = 3813; genome coverage > 60%), which formed several tightly clustered groups, only 46 representative sequences were retained to avoid over-representation. Sequences that did not form clusters were also retained to preserve unique genetic diversity.

Subsequently, these representative sequences were randomly selected from each cluster to minimize the impact of sampling bias while ensuring the maintenance of phylogenetic diversity. The resulting datasets were analyzed separately for the two major CHIKV lineages: ECSA and Asian. Multiple sequence alignments were performed for each lineage using MAFFT, and these alignments were manually inspected for accuracy. The temporal signal was evaluated using TempEst v1.5.3 (developed by Andrew Rambaut, University of Edinburgh, UK), and sequences that deviated significantly from a molecular clock were excluded from further analysis. Recombination screening was performed using RDP4 (developed by Darren P. Martin, University of Cape Town, South Africa) and SimPlot v3.5.1 (developed by Stuart C. Ray, Johns Hopkins University School of Medicine, Baltimore, MD, USA), and no significant recombination events were detected. Final datasets comprised 312 ECSA and 135 Asian lineage genomes.

Bayesian phylogenetic and phylogeographic inference for each lineage was performed using BEAST (v1.10.5) [19]. The most suitable nucleotide substitution models, as determined by ModelFinder in IQ-TREE under the Bayesian Information Criterion, were GTR + F + I + G4 for the ECSA lineage and GTR + F + G4 for the Asian lineage. The molecular clock was relaxed, and a constant size tree prior was applied. Given that the primary objective was to infer spatial diffusion rather than population demographic dynamics, a parsimonious tree prior was employed to reduce model complexity. The geographic diffusion process was modeled as a discrete trait with an asymmetric transition rate matrix. Bayesian stochastic search variable selection (BSSVS) was employed to identify well-supported dispersal routes. For the ECSA lineage, two independent Markov chain Monte Carlo (MCMC) runs were performed for 300 million generations each, with sampling every 20,000 generations. For the Asian lineage, two independent MCMC runs of 150 million generations each were conducted, with a sampling frequency of 15,000 generations, to assess convergence and reproducibility. In all analyses, the initial 10% of samples were discarded as burn-in.

The convergence and mixing processes were evaluated using Tracer v1.6 [19], ensuring that effective sample sizes exceeded 200 for all key parameters. Convergence between independent runs was further assessed by comparing marginal posterior distributions and calculating the potential scale reduction factor (PSRF), with results reported in Tables S1 and S2. The posterior tree distributions were summarized using TreeAnnotator (v1.10.4) [19] to generate maximum clade credibility (MCC) trees, which were then visualized with FigTree, with a focus on lineages associated with outbreaks in China. The spatial dissemination pathways were reconstructed by analyzing the inferred geographic states of the nodes across the MCC trees and posterior tree sets. The statistical support for individual dispersal routes was quantified using SPREAD3 v0.9.6 (developed by Filip Bielejec et al., KU Leuven, Belgium), which estimates transition rates between geographic regions under the BSSVS framework and reports the Bayes factor (BF) [20]. Dispersal routes with BF > 3 were considered substantially supported. Furthermore, Markov jump counts were extracted to quantify the expected number of viral migration events between geographic regions over the evolutionary history. All data processing, diffusion network reconstruction, and spatiotemporal visualizations were performed in R v4.1.2 (R Core Team, R Foundation for Statistical Computing, Vienna, Austria).

## Results

### Temporal and spatial patterns of imported and local chikungunya cases in China

**Fig. 1:**
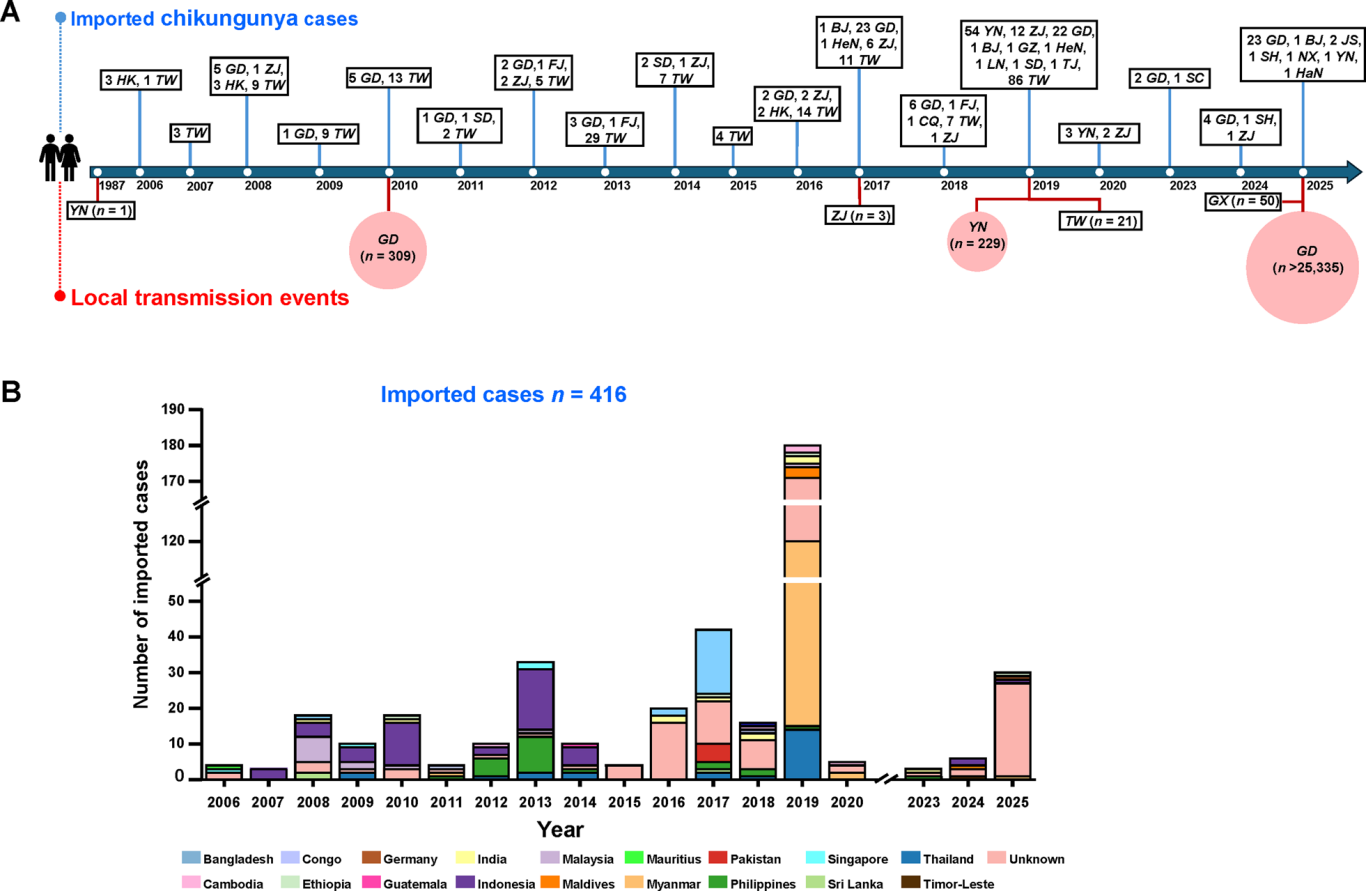
Temporal and spatial distribution of imported and local chikungunya cases in China. **A** Timeline of imported chikungunya cases and local transmission events in China from 2006 to 2025. **B** Annual distribution of imported CHIKV cases in China (*n* = 416) by country of origin, from 2006 to 2025. Abbreviations for Chinese provinces include: *BJ* Beijing, *CQ* Chongqing, *FJ* Fujian, *GD* Guangdong, *GZ* Guizhou, *HaN* Hainan, *HeN* Henan, *HK* Hong Kong, *JS* Jiangsu, *LN* Liaoning, *NX* Ningxia, *SC* Sichuan, *SD* Shandong, *SH* Shanghai, *TJ* Tianjin, *TW* Taiwan, *YN* Yunnan, *ZJ* Zhejiang

A comprehensive collection of epidemiological data was obtained from 54 publications that were identified through database searches (Table S3). The contemporary period of CHIKV introduction into China commenced in 2006, with the initial clearly documented cases reported in Hong Kong and Taiwan. The first documented instance of the disease on the Chinese mainland occurred in 2008. After that period, imported cases were documented on an annual basis, except a temporary cessation in 2021–2022 that was attributed to the implementation of travel restrictions in response to the ongoing pandemic (Fig. 1A). By December 2025, a cumulative total of 416 imported CHIKV cases had been reported in China, including cases originating from 19 identified countries and 135 cases with unknown country of origin (Table 1). The cases were geographically concentrated in border and coastal provincial-level administrative divisions (PLADs). Taiwan reported the highest number of cases (*n* = 200), followed by Guangdong (*n* = 99), Yunnan (*n* = 58), and Zhejiang (*n* = 28). All other provinces reported several cases that did not exceed ten (Table 2). In 2019, the number of imports reached 180, the highest in a single year. A considerable proportion of these cases, 58% (105/180), originated in Myanmar. Other noteworthy annual peaks emerged in 2017 (*n* = 42), 2013 (*n* = 33), and 2025 (*n* = 30) (Fig. 1B). Among the cases with a traceable origin, Myanmar was identified as the leading source country (*n* = 109), followed by Indonesia (*n* = 50), Thailand (*n* = 24), the Philippines (*n* = 23), and Bangladesh (*n* = 21) (Table 1).

**Table 1:** Source countries of imported CHIKV cases in China (*n* = 416)

Source country	Imported cases (*n*)
Bangladesh	21
Cambodia	5
Republic of the Congo	2
Ethiopia	4
Germany	1
Guatemala	1
India	10
Indonesia	50
Kenya	1
Malaysia	12
Maldives	4
Mauritius	1
Myanmar	109
Pakistan	5
Philippines	23
Singapore	4
Sri Lanka	3
Thailand	24
Timor-Leste	1
Unknown	135
Total	416

**Table 2:** Reporting provincial-level administrative divisions (PLADs) of imported CHIKV cases in China (*n* = 416)

Reporting PLAD	Imported cases (*n*)
Beijing	3
Chongqing	1
Fujian	3
Guangdong	99
Guizhou	1
Hainan	1
Henan	2
Hong Kong	8
Jiangsu	2
Liaoning	1
Ningxia	1
Shandong	4
Shanghai	2
Sichuan	1
Taiwan	200
Tianjin	1
Yunnan	58
Zhejiang	28
Total	416

The first documented instance of CHIKV isolation occurred in 1987 in Yunnan Province, China, from a patient. However, no genomic sequence from this isolate is publicly available, and therefore it was not included in subsequent analyses. The initial outbreak was documented in 2010 in Guangdong Province, where 309 cases were confirmed. In 2017, three cases of limited local transmission were identified, with the causative agent traced to an importation from Bangladesh. Subsequent to this, local outbreaks were documented in Yunnan (229 cases) and Taiwan (21 cases) in the latter half of 2019. The most significant outbreak occurred in 2025 in Guangdong Province, with a total of 25,335 confirmed cases (Fig. 1A). The outbreak exhibited a high degree of concentration in the cities of Jiangmen and Foshan, accounting for over 20,000 cases. The outbreak also spread to the neighboring PLAD of Guangxi, where 50 cases were reported. A total of 25,948 local cases have been documented in China, with Guangdong Province demonstrating the highest incidence of infection (Table 3).

**Table 3:** Distribution of local CHIKV outbreak cases by region in China

Local outbreak region (Province/City)	Reported CHIKV cases (local)
Guangdong_Shanwei	36
Guangdong_Heyuan	40
Guangdong_Maoming	40
Guangdong_Yunfu	44
Guangdong_Meizhou	78
Guangdong_Shaoguan	87
Guangdong_Huizhou	114
Guangdong_Yangjiang	117
Guangdong_Qingyuan	133
Guangdong_Jieyang	167
Guangdong_Zhanjiang	195
Guangdong_Zhongshan	208
Guangdong_Chaozhou	220
Guangdong_Shantou	225
Guangdong_Zhuhai	256
Guangdong_Zhaoqing	278
Guangdong_Dongguan	484
Guangdong_Shenzhen	743
Guangdong_Guangzhou	1292
Guangdong_Jiangmen	10034
Guangdong_Foshan	10853
Yunnan_Ruili	99
Yunnan_Xishuangbanna	88
Yunnan_Lincang	43
Zhejiang_Quzhou	3
Guangxi_Nanning	50
Taiwan	21
Total	25948

### Lineage composition of imported and locally circulating CHIKV in China

**Fig. 2:**
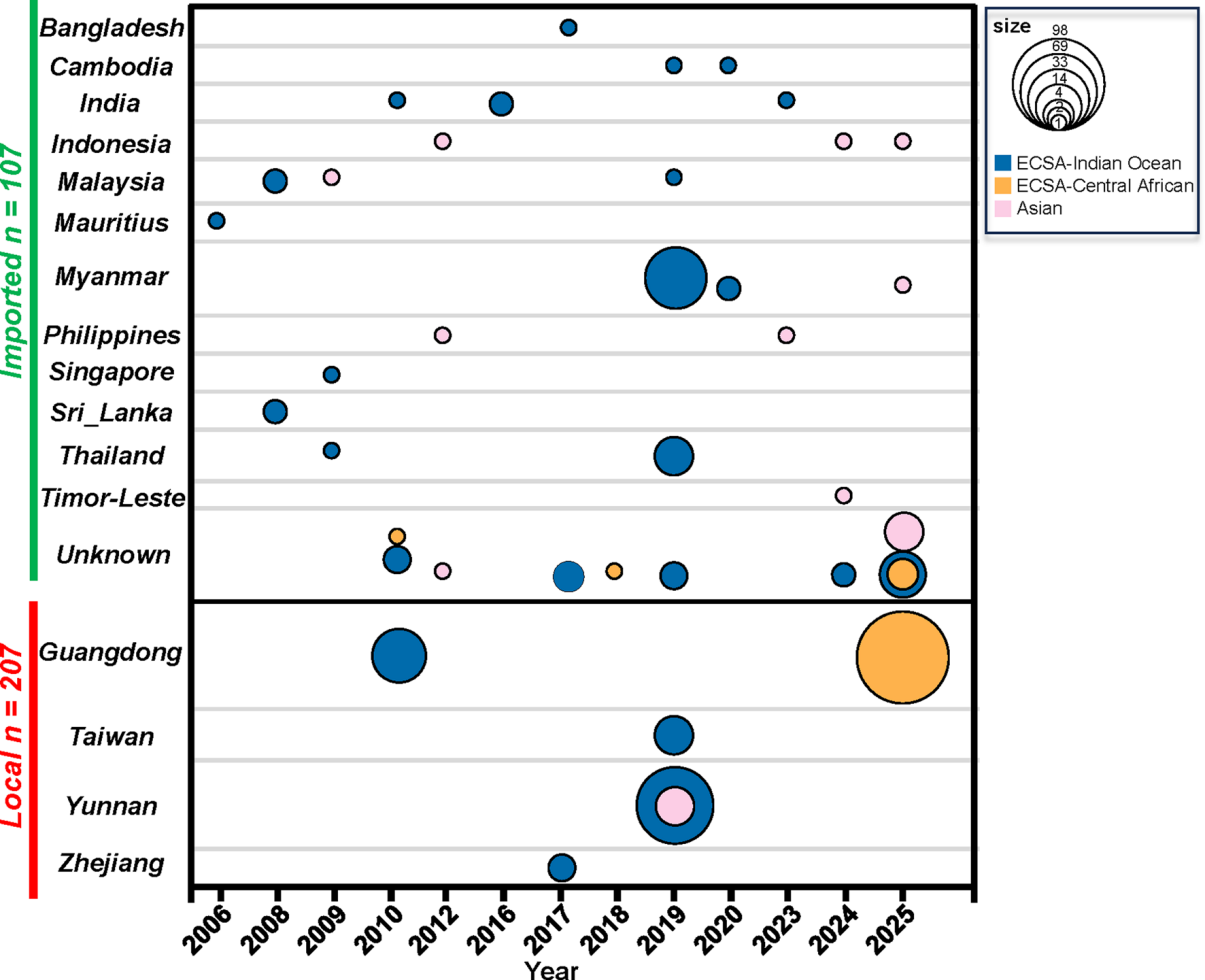
Spatiotemporal distribution of viral genotypes in imported and locally acquired chikungunya cases in China, 2006–2025. Bubble size is proportional to the number of cases, with larger bubbles indicating higher case numbers. Colors represent different lineages/branches of the virus

A subsequent analysis of the 314 CHIKV sequences from China revealed the presence of two predominant lineages: the ECSA lineage, which constituted 92.0% (289/314) of the sequences, and the Asian lineage, comprising 8.0% (25/314) (Fig. S2). Comprehensive metadata of all sequences are available in the supplementary tables 4 and 5. The ECSA lineage was further divided into two clades: the Central African clade, which constituted 58.9% (185/314) of the sequences, and the IOL, representing 33.1% (104/314). A statistically significant difference in lineage distribution was observed between imported cases (*n* = 107) and locally acquired cases (*n* = 207) (χ^2^ test, χ^2^ = 31.84, *P* < 0.001). Among the imported cases, the IOL clade was predominant, comprising 78.5% (84/107), followed by the Asian lineage at 15.9% (17/107), while the Central African clade accounted for only a small fraction, at 5.6% (6/107). In contrast, among locally acquired cases, the IOL clade was identified in 48.8% (101/207) of instances, and the Central African clade was detected at a comparable rate of 47.3% (98/207). The Asian lineage was identified in a lower percentage of cases, at 3.9% (8/207) (Fig. 2). It should be noted that these proportions reflect the phylogenetic diversity within the curated dataset rather than the true epidemiological prevalence, since sequencing coverage does not necessarily correspond to the number of reported clinical cases. A comparison between the number of reported clinical cases and available sequences for each outbreak is provided in Table S6.

The patterns of local outbreaks have undergone substantial changes over time. The outbreaks that occurred in 2010 and 2017 were primarily caused by the ECSA-IOL clade. However, during the 2019 outbreak in Yunnan Province, there was co-circulation of both the ECSA-IOL and Asian lineages. A notable example is the large-scale outbreak in Guangdong Province in 2025, which was primarily attributed to the ECSA-Central African clade. The temporal coincidence of each local outbreak with an increase in imported cases related to the corresponding lineage suggests a potential causative relationship. Furthermore, the lineage responsible for each outbreak aligned with the predominant lineage found among imported cases just before the outbreak occurred (Fig. 2).

### Bayesian phylogenetic analyses

**Fig. 3:**
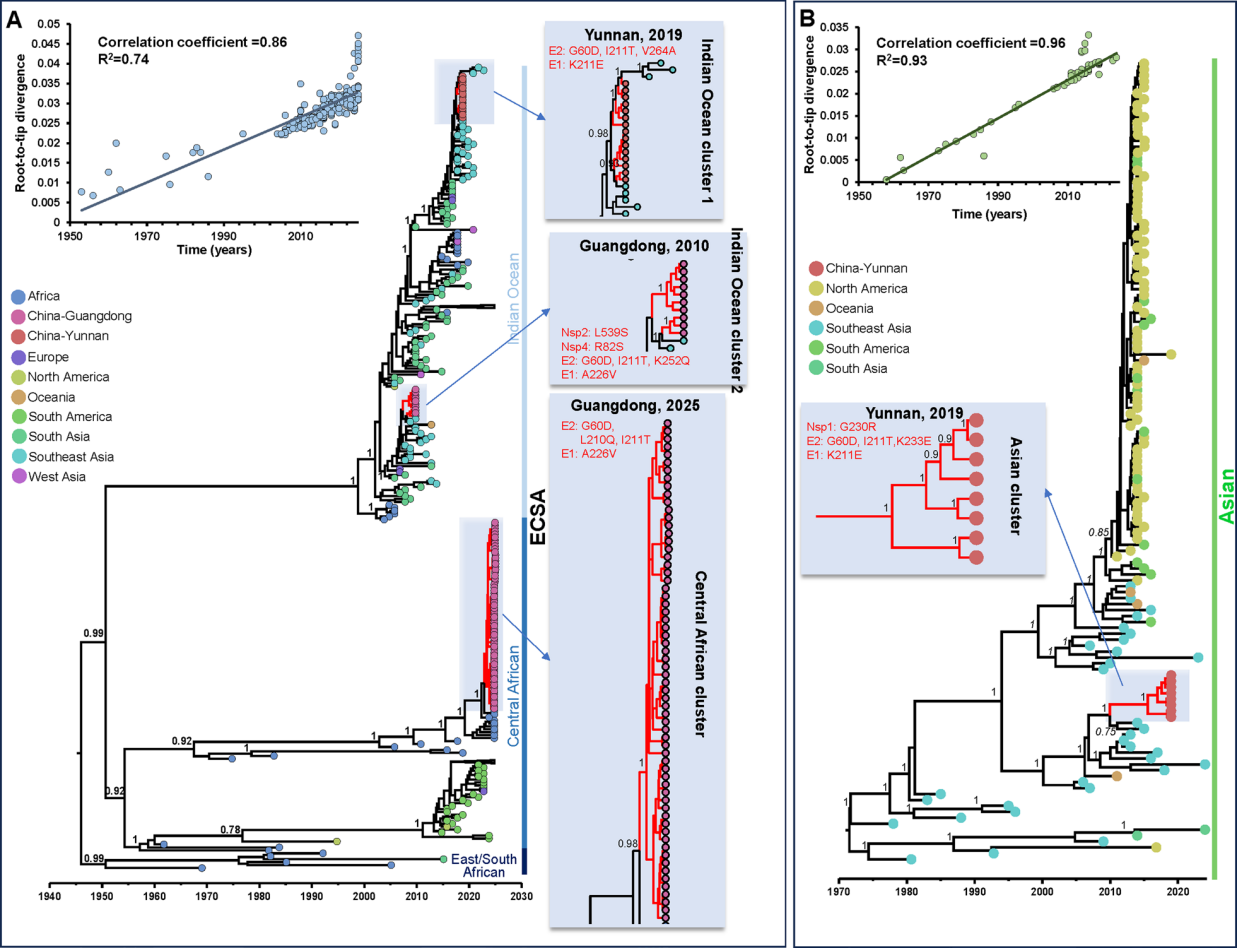
Bayesian time-scaled phylogenetic tree revealing four clusters responsible for locally acquired chikungunya outbreaks in China. Branch posterior probabilities associated with geographic transmission are annotated on the tree. **A** Time-scaled phylogenetic tree of 312 ECSA lineage sequences (BEAST 1.10.5) revealing three clusters: ECSA-Indian Ocean cluster 1 (Yunnan, 2019), ECSA-Indian Ocean cluster 2 (Guangdong, 2010), and the Central African cluster (Guangdong, 2025). **B** Time-scaled phylogenetic tree of 135 Asian lineage sequences identifying Asian Cluster (Yunnan, 2019). The upper left corner of **A** and **B** displays linear regression plots of genetic distance versus time (years) for two lineages, showing significant correlations (ECSA: correlation coefficient = 0.86, R^2^ = 0.74; Asian: correlation coefficient = 0.96, R^2^ = 0.93)

The ECSA and Asian lineage datasets exhibited robust temporal signals in their analysis. The application of root-to-tip regression analysis to the ECSA dataset yielded a correlation coefficient of 0.86 and an R^2^ of 0.74. In contrast, the Asian dataset exhibited a correlation coefficient of 0.96 and an R^2^ of 0.93 (Fig. 3). Bayesian inference using a relaxed molecular clock model estimated the mean evolutionary rate of the ECSA lineage at 7.87 × 10⁻^4^ substitutions per site per year (with a 95% highest posterior density [HPD] interval of 6.48 × 10⁻^4^ to 9.33 × 10⁻^4^). In contrast, the Asian lineage exhibited a lower mean evolutionary rate of 3.76 × 10⁻^4^ substitutions per site per year (95% HPD: 2.91 × 10⁻^4^ to 4.71 × 10⁻^4^). Time-scaled phylogenetic analysis identified four independent transmission clusters associated with local outbreaks in China (Fig. 3). These included IOL Cluster 1 and an Asian cluster related to the 2019 outbreak in Yunnan, IOL Cluster 2 linked to the 2010 outbreak in Guangdong, and a Central African cluster responsible for the 2025 outbreak in Guangdong. An analysis of the identified transmission clusters revealed the presence of mutations that have been previously documented in circulating CHIKV strains [21]. The estimated time to the most recent common ancestor (tMRCA) was determined to be May 2017 (95% HPD: 2017.0–2017.9) for IOL Cluster 1; March 2007 (95% HPD: June 2006–January 2008) for IOL Cluster 2; March 2023 (95% HPD: 2022.8–2023.9) for the Central African cluster; and May 2015 (95% HPD: 2014.0–2017.0) for the Asian cluster (Fig. 3).

### Phylogeographic reconstruction and statistically supported dispersal routes of CHIKV in China

**Fig. 4:**
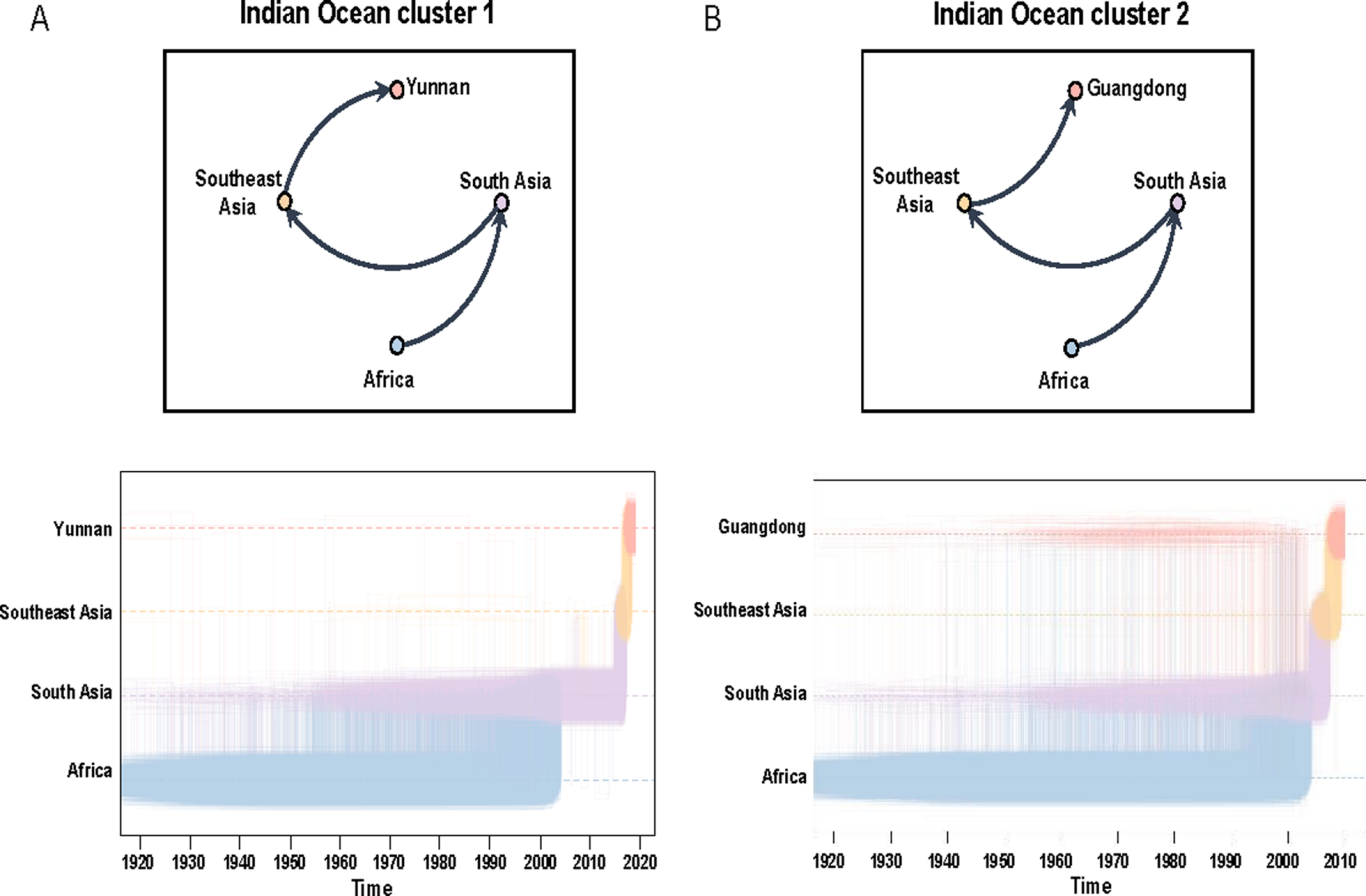
Global phylogeographic histories of ECSA-Indian Ocean clusters associated with locally acquired chikungunya transmission in China. **A** Indian Ocean cluster 1 and **B** Indian Ocean cluster 2. Arrows in the upper panels indicate inferred geographic transitions between ancestral locations reconstructed from the MCC tree and the corresponding Chinese transmission clusters. The lower panels summarize Markov jump histories inferred from the posterior phylogeographic reconstruction. The figure displays inferred timepoints for Markov jumps, with vertical lines representing geographic transitions and horizontal lines indicating periods where lineages remained stationary at a single location. Densely clustered lines indicate higher certainty for transmission events at specific dates, while sparse lines suggest lower certainty

**Fig. 5:**
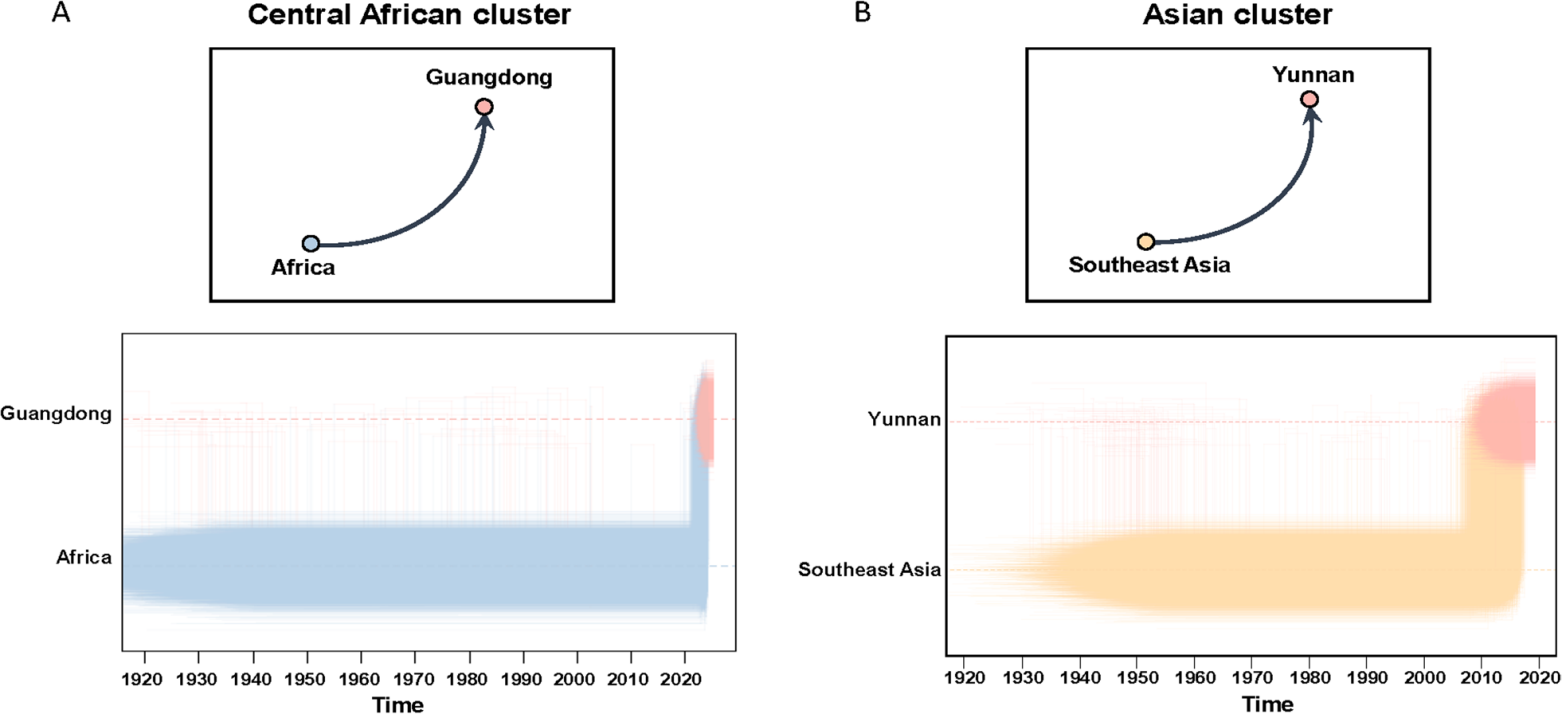
Global phylogeographic histories of ECSA-Central African and Asian clusters associated with locally acquired chikungunya transmission in China. **A** Central African cluster and **B** Asian cluster. Arrows in the upper panels indicate inferred geographic transitions between ancestral locations reconstructed from the MCC tree and the corresponding Chinese transmission clusters. The lower panels summarize Markov jump histories inferred from the posterior phylogeographic reconstruction. The figure displays inferred timepoints for Markov jumps, with vertical lines representing geographic transitions and horizontal lines indicating periods where lineages remained stationary at a single location. Densely clustered lines indicate higher certainty for transmission events at specific dates, while sparse lines suggest lower certainty

A comprehensive investigation into the global-scale phylogeographic patterns exhibited by the major CHIKV genotypes has been conducted. Migration pathway diagrams for the ECSA and Asian genotypes are provided in Supplementary Figs. 3 and 4, respectively. These diagrams illustrate extensive intercontinental and regional diffusion patterns. The origins and dissemination routes of four primary transmission clusters were subsequently traced using discrete phylogeographic reconstruction. In order to analyze the specific movement dynamics of each cluster, the Markov jump histories among locations were summarized. This facilitated the identification of major transmission trajectories. The two IOL clusters (1 and 2) are believed to have originated in either Africa or South Asia. These clusters subsequently disseminated to Southeast Asia in the early twenty-first century, and were introduced into the Yunnan and Guangdong provinces of China (Fig. 4). In contrast, the Central African cluster appears to have been predominantly circulating in Africa. The analysis indicates that, in the early twenty-first century, there is a history of jumps between this cluster and the Guangdong samples, suggesting a possible introduction from Africa into Guangdong. The Asian cluster exhibited a distinct pattern, with sustained circulation in Southeast Asia during the twentieth century, followed by its introduction into Yunnan Province (Fig. 5).

**Fig. 6:**
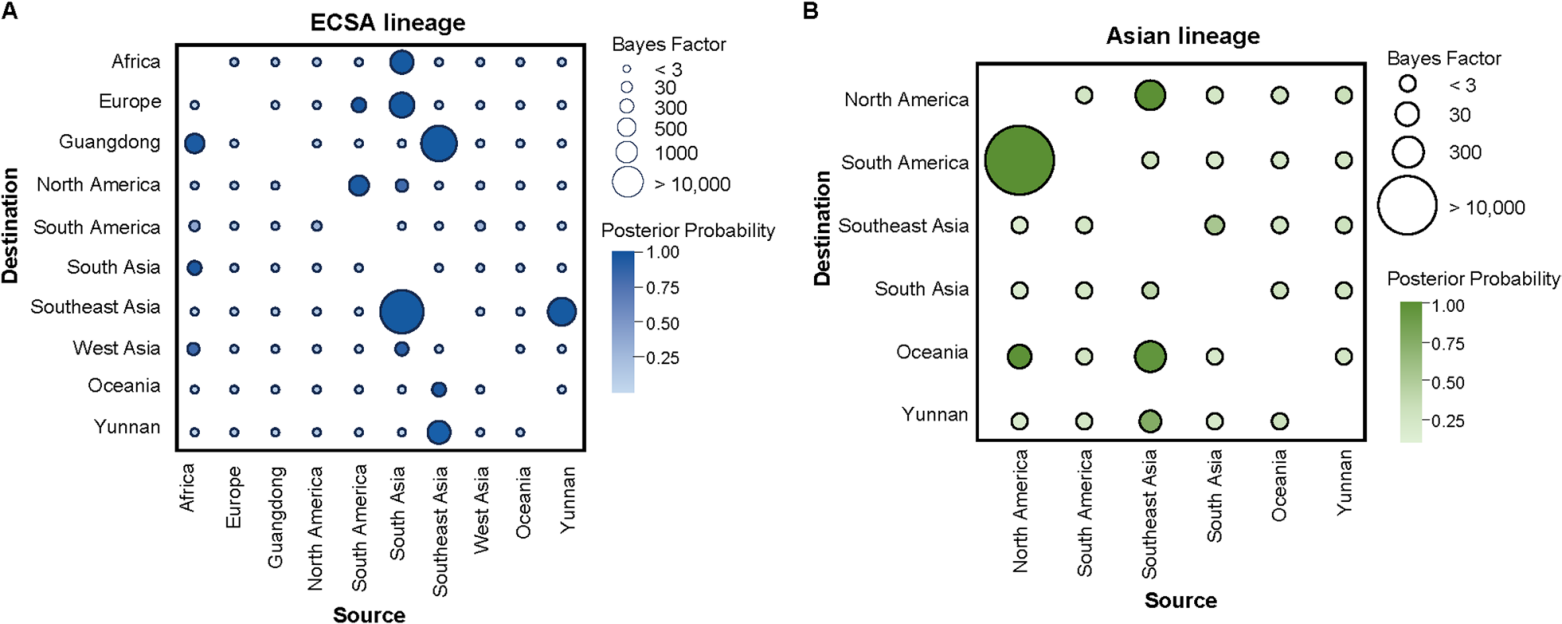
Global transmission support for ECSA and Asian lineages of CHIKV based on systematic geographic analysis. **A** ECSA lineage of CHIKV and **B** Asian lineage of CHIKV. The matrix displays PP of transmission between source and destination locations, with colors indicating the magnitude of PP and bubble size representing the strength of BF for transmission

Our analysis of the ECSA genotype revealed the main dissemination routes within regions, including Africa, South Asia, Southeast Asia, as well as within China (specifically Yunnan and Guangdong provinces). Several key dissemination routes were strongly supported by high BFs and posterior probabilities (PPs). Specifically, the Africa-to-South Asia route had a BF of 166.33 and a PP of 0.95, the South Asia-to-Southeast Asia route had a BF of 223,684.28 and a PP of 1.00, the Africa-to-Guangdong route had a BF of 677.88 and a PP of 0.98, the Southeast Asia-to-Yunnan route had a BF of 1503.15 and a PP of 0.99, and the Southeast Asia-to-Guangdong route had a BF of 55,914.85 and a PP of 0.99. For the Asian genotype, the analysis revealed multiple dissemination routes. The Southeast Asia-to-Yunnan route was supported by both BF and PP (BF = 14.06, PP = 0.76) (Fig. 6) (Table S7).

## Discussion

This study integrated epidemiological data with viral genomic information to comprehensively investigate the characteristics of CHIKV importation, transmission, and evolution in China. The findings indicated a shift in the epidemiological landscape of CHIKV in China, transitioning from sporadic imported cases to multiple localized outbreaks, ultimately resulting in the most significant recorded epidemic in Guangdong Province in 2025. Local CHIKV transmission in China is driven not by sustained circulation of a single lineage, but by multiple independent introductions from distinct geographic origins. This pattern indicates a significant and increasing risk of CHIKV importation and local outbreaks in China.

Since 2006, the number of imported CHIKV cases in China has closely matched the volume of international travel. The distribution of these cases has been primarily concentrated in coastal and border PLADs that have frequent international exchanges, such as Guangdong [22], Zhejiang [23], Taiwan [24], and Yunnan [25]. A noteworthy observation is the temporal coincidence between local outbreaks and the peak activity period of the primary mosquito vectors in these regions, which occurred between July and October [13–15]. The findings indicate that a combination of sustained viral importation, seasonal peaks in vector density, and favorable climatic conditions creates a significant risk framework for establishing local transmission chains. Subsequent phylogenetic analysis revealed discrepancies in lineage composition between imported cases and cases acquired locally. The majority of imported cases were classified as belonging to the ECSA-IOL clade, while the proportion of locally acquired cases was nearly equal to both the ECSA-IOL and ECSA-Central African clades. These findings suggest that local outbreaks are influenced by various factors, including the source of importation and the virus's ability to adapt to the local environment, rather than being solely determined by the most frequently imported lineage. In the regions of Guangdong and Taiwan, importation patterns exhibit an "airport-portal" model, distinguished by relatively concentrated sources [26]. In contrast, Yunnan exemplifies a "land border portal" model, which involves a more complex situation with multiple sources and lineages [27]. For instance, both the ECSA-IOL and Asian lineages were observed to co-circulate in Yunnan during 2019 [14, 28]. This scenario underscores the imperative for enhanced surveillance and early warning capabilities.

The lineages responsible for the Guangdong outbreaks in 2010 and 2025 were different, specifically ECSA-IOL and ECSA-Central African, respectively. However, viruses from both outbreaks carried the classic adaptive mutation, E1-A226V. A series of earlier studies has established a correlation between this mutation and the enhanced adaptation of the CHIKV to the mosquito species *Ae. albopictus* [29]. The recurrent appearance of this mutation in the two notable outbreaks in Guangdong indicates a plausible association with elevated local transmission. In addition to E1-A226V, several other mutations identified in this study have been functionally associated with vector adaptation. The ECSA-IOL lineage responsible for the 2019 Yunnan outbreak concurrently harbored the E1-K211E and E2-V264A mutations, a combination that has been shown to enhance viral dissemination in *Ae. aegypti*, increasing both midgut infection and transmission efficiency [21, 30]. Mutations such as E2-G60D and E2-I211T, which were repeatedly detected across different outbreaks and lineages in China, have been demonstrated to enhance infectivity in both *Ae. aegypti* and *Ae. albopictus*. Notably, the effect of E2-G60D is modulated by the genetic background at the E1-226 site: in the presence of E1-226V, E2-G60D may act synergistically with E2-I211T to restore or enhance vector infectivity [31]. In addition, the E2-L210Q mutation identified in the 2025 Guangdong outbreak has been associated with adaptation to *Ae. albopictus*, particularly through enhanced midgut infection and dissemination efficiency [32]. The adaptive significance of these mutations should be interpreted in the context of the geographic distribution of major mosquito vectors in China. *Ae. aegypti* is primarily concentrated in Guangdong, Hainan, and Yunnan Provinces, whereas *Ae. albopictus* has a much broader distribution, ranging from southern regions to as far north as Shandong [33]. The sympatric distribution of these two vector species in southern China may create a selective environment that favors viral variants with broader or enhanced vector adaptability. Taken together, the interplay between viral genetic adaptation and the regional distribution of mosquito vectors likely plays a critical role in shaping CHIKV transmission dynamics in southern China.

Bayesian time-scaled phylogenetic analysis identified four independent local transmission clusters of the CHIKV. The time to tMRCA for each cluster is prior to the associated outbreak. However, there was no evidence of long-term, sustained circulation of the virus. These findings support the conclusion that the spread of CHIKV in China is primarily driven by repeated independent introductions followed by short-term local diffusion, rather than a stable endemic presence. This pattern is in stark contrast to regions of high CHIKV endemicity, such as Southeast Asia [34]. These findings suggest that China is still in the early stages of CHIKV expansion. Early detection and intervention at the point of importation could help prevent the virus from establishing persistent local transmission chains.

Discrete phylogeographic analysis in this study suggests that the 2010 Guangdong outbreak originated from Southeast Asia, the 2019 Yunnan outbreak similarly originated from Southeast Asia, whereas the 2025 Guangdong outbreak may be associated with an introduction from Africa. The 2010 Guangdong strains showed high genetic similarity to strains from Thailand, Malaysia, and Singapore [13]. At that time, Southeast Asia was experiencing widespread chikungunya outbreaks, with large numbers of cases reported in countries including Malaysia, Singapore, Thailand, and Indonesia [35]. As a major hub for foreign trade, Guangdong maintains high levels of human mobility with Southeast Asia through the Canton Fair, dense flight networks, and bilateral trade, providing a plausible pathway for viral introduction. Similarly, the ECSA-IOL strains from the 2019 Yunnan outbreak were highly similar to those from Thailand [14]. During the same period, Thailand reported approximately 28,000 cases [36], and outbreaks were also occurring in Myanmar. Cross-border movement in border regions such as Ruili (with over 49,000 daily crossings) and Xishuangbanna, characterized by frequent tourism, trade, and local exchanges, likely facilitated viral introduction [37]. Notably, the strains from the 2025 Guangdong outbreak formed the closest phylogenetic cluster with those from Réunion Island [38, 39], where a large-scale epidemic occurred during 2024–2025. Historically, the Chinese community in Réunion Island originates largely from migration waves from Guangdong in the nineteenth century [40]. These long-standing familial and cultural ties sustain frequent travel between the two regions and may provide potential pathways for unmonitored viral introductions. Consequently, the province of Guangdong may encounter import pressure from Southeast Asia as well as from less predictable, potential long-distance intercontinental introductions [35].

Non-classical CHIKV lineages can spread rapidly and extensively when introduced into ecologically suitable environments, especially under conditions of high human mobility. Previous studies have shown that CHIKV transmission is strongly influenced by a combination of climatic conditions, vector density, and population-related factors. For example, modelling of the 2019 outbreak in Yunnan identified imported cases, population density, and precipitation-related factors as key determinants of local transmission [25]. Similarly, a 2025 study in Guangdong demonstrated that sustained high temperature and precipitation promoted mosquito proliferation and increased transmission risk [38]. These findings collectively suggest that the rapid spread of non‑classical lineages is facilitated by the interplay of climatic, ecological, and human‑related factors, though the relative contribution of each factor may vary across regions. Further quantifying the relative contributions of these factors will require future studies that integrate geographic, climatic, and human mobility data.

Additionally, the co-circulation of other arboviruses in southern China is concerning. Previous studies have reported CHIKV-dengue virus (DENV) coinfections, with the largest number of cases occurring during the 2019 outbreak in Xishuangbanna, Yunnan Province, where 86 cases were identified [28]. There have also been sporadic reports of coinfections, including a CHIKV-DENV coinfection in an imported case from Southeast Asia reported in Guangdong in 2016 [22], and a CHIKV-Zika virus coinfection in an imported case from the Philippines in 2019 [41]. Furthermore, CHIKV and Japanese encephalitis virus have been detected in the same mosquito vector, *Culex tritaeniorhynchus*, in Honghe, Yunnan Province [42]. These observations suggest that CHIKV transmission in southern China may occur in settings where multiple arboviruses co-circulate. Therefore, future surveillance and outbreak investigations should screen simultaneously for CHIKV and other relevant mosquito-borne viruses, particularly DENV.

This study has several limitations. First, although a relatively large number of CHIKV genomes from China were included, the availability of genomic data does not necessarily correspond to the number of reported clinical cases, and sequencing coverage varied across outbreaks in China. This may have resulted in an underrepresentation of large outbreaks and a potential underestimation of transmission links or omission of some outbreak sources. Second, the stratified subsampling strategy reduced the global dataset from 5868 to 447 sequences, which may limit the spatial resolution of the phylogeographic analysis. Although necessary for computational feasibility, this approach may obscure finer-scale transmission dynamics and potential genetic links between specific regions. Third, the study relies on viral sequences from human clinical cases. Consequently, the paucity of contemporary genomic data from local mosquito vectors limits our ability to directly validate viral adaptive changes within the human-mosquito-human transmission cycle. Fourth, environmental and population-related drivers of CHIKV transmission were not quantitatively assessed. As a result, their relative contributions to transmission dynamics could not be determined.

## Conclusions

In conclusion, this study highlights the increasing risk of CHIKV importation and localized outbreaks in China, driven by multiple independent introductions and the virus's ability to adapt to local conditions. As the risk of China importing CHIKV and experiencing major outbreaks increases, it is essential to implement a proactive and integrated defense strategy. It is recommended that integrated surveillance systems be deployed in key provinces, such as Yunnan and Guangdong, with the objective of facilitating the early genomic identification of threatening variants. Additionally, it is recommended that risk assessment frameworks be expanded to monitor global spread patterns, with a particular focus on the African continent. The implementation of sustained targeted vector control and community outreach initiatives is also imperative. This comprehensive approach is vital for interrupting transmission chains and preventing future epidemics.

## Supplementary Information

Supplementary material 1: Table S1. PSRF values for parameters estimated from Bayesian MCMC analyses of the ECSA lineage dataset.

Supplementary material 2: Table S2. PSRF values for parameters estimated from Bayesian MCMC analyses of the Asian lineage dataset.

Supplementary material 3: Table S3. Summary of reported chikungunya cases in China.

Supplementary material 4: Table S4. GenBank, GISAID, and GenBase accession numbers of CHIKV sequences used in this study and amino acid variation at positively selected sites in E1 and E2 proteins (imported cases).

Supplementary material 5: Table S5. GenBank and GenBase accession numbers of CHIKV sequences used in this study and amino acid variation at positively selected sites in E1 and E2 proteins (local transmission cases).

Supplementary material 6: Table S6. Annual number of reported CHIKV cases and available sequences in China, 1987–2025.

Supplementary material 7: Table S7. Confirmed routes of cross-regional transmission for each CHIKV genotype.

Supplementary material 8: Fig S1. Workflow for data collection, sequence processing, and phylogenetic/phylogeographic analyses of CHIKV in China.

Supplementary material 9: Fig S2. Maximum likelihood phylogenetic analysis reveals indigenous and imported chikungunya virus lineages/branches in China.

Supplementary material 10: Fig S3. The Bayesian discrete geographic tree reveals the global transmission pathways of the ECSA lineage of chikungunya virus.

Supplementary material 11: Fig S4. The Bayesian discrete geographic tree reveals the global transmission pathways of the Asian lineage of chikungunya virus.

## Data Availability

All data used for the analyses in this study are provided in the Supplementary Information files.

## Abbreviations

BF: Bayes factors
BSSVS: Bayesian stochastic search variable selection
CHIKV: Chikungunya virus
CNKI: China National Knowledge Infrastructure
DENV: Dengue virus
ECSA: East/Central/South African
GenBase: China National Genomics Data Center
GISAID: Global Initiative on Sharing All Influenza Data
IOL: Indian Ocean clade
MCC: Maximum clade credibility
MCMC: Markov chain Monte Carlo
NCBI: National Center for Biotechnology Information
PP: Posterior probabilities
PSRF: Potential scale reduction factor
tMRCA: Time to the most recent common ancestor
