# Supplementary material for: Genomic epidemiology reveals the origins and transmission dynamics of chikungunya virus in China
Source: Infect Dis Poverty. 2026 Jun 4;15:64. doi: 10.1186/s40249-026-01465-2 (PMC13234983; doi:10.1186/s40249-026-01465-2)
Supplement: Supplementary file 1 — Supplementary material 1: Table S1. PSRF values for parameters estimated from Bayesian MCMC analyses of the ECSA lineage dataset. [file 40249_2026_1465_MOESM1_ESM.docx]

**Fig. S3 The Bayesian discrete geographic tree reveals the global transmission pathways of the ECSA lineage of chikungunya virus.**

**
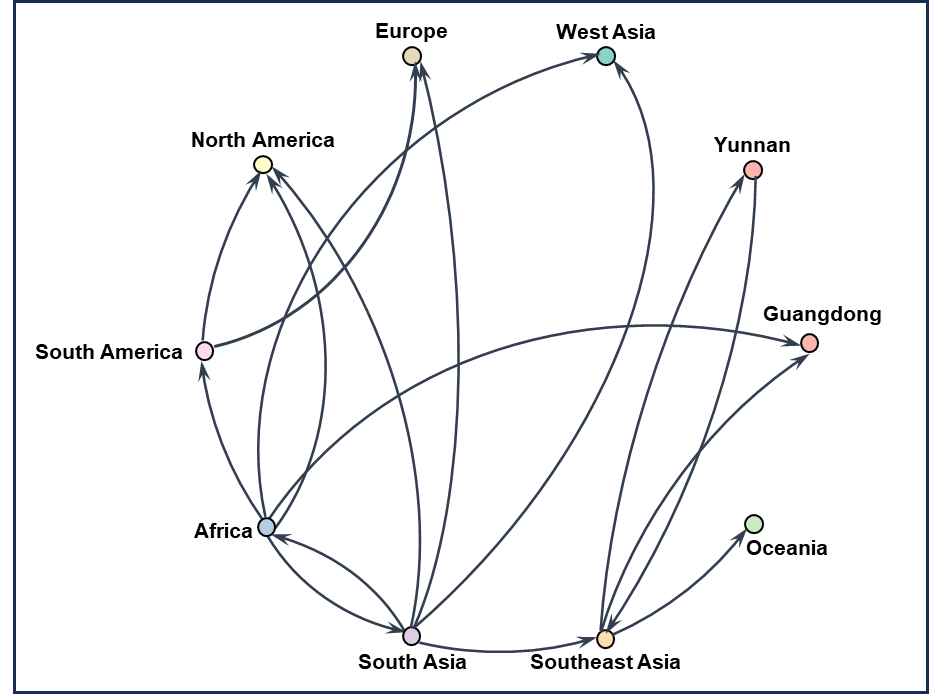
**
