# Supplementary material for: Genomic epidemiology reveals the origins and transmission dynamics of chikungunya virus in China
Source: Infect Dis Poverty. 2026 Jun 4;15:64. doi: 10.1186/s40249-026-01465-2 (PMC13234983; doi:10.1186/s40249-026-01465-2)
Supplement: Supplementary file 3 — Supplementary material 3: Table S3. Summary of reported chikungunya cases in China. [file 40249_2026_1465_MOESM3_ESM.docx]

**Table S1** PSRF values for parameters estimated from Bayesian MCMC analyses of the ECSA lineage dataset.

| Parameter | PSRF | Upper_CI |
| --- | --- | --- |
| location.indicators.South_Asia.Europe | 1.20036759714843 | 1.23838322808972 |
| location.indicators.South_Asia.Africa | 1.05105827849133 | 1.05299604367658 |
| location.indicators.Southeast_Asia.Yunnan | 1.02536256441218 | 1.0271225583642 |
| treeLength | 1.02355341238536 | 1.10781916401109 |
| default.covariance | 1.0227313866608 | 1.04618187448536 |
| default.meanRate | 1.02223850335216 | 1.10243626334467 |
| location.indicators.Africa.Guangdong | 1.02207000843755 | 1.02529436712416 |
| location.indicators.South_America.North_America | 1.02207000843755 | 1.02529436712416 |
| coalescent | 1.01618585220377 | 1.07427236091677 |
| constant.popSize | 1.01377232700007 | 1.06199752012084 |
| default.ucld.mean | 1.01245840482139 | 1.0574656906399 |
| location.rates.Yunnan.Africa | 1.01213794748337 | 1.0237735479017 |
| prior | 1.01052289357713 | 1.04799356653213 |
| location.rates.North_America.Europe | 1.00988590632309 | 1.02643387083852 |
| location.indicators.Yunnan.Southeast_Asia | 1.00970323335601 | 1.01003905806883 |
| tmrca(untitled2) | 1.00891820475417 | 1.04001879770907 |
| age(untitled2) | 1.00891820474376 | 1.04001879769856 |
| location.indicators.North_America.West_Asia | 1.00876143795188 | 1.02282966722876 |
| location.indicators.South_America.Yunnan | 1.0081422346359 | 1.01278489883592 |
| location.rates.Southeast_Asia.West_Asia | 1.00647240267503 | 1.00814868456572 |
| location.indicators.South_Asia.Oceania | 1.00626751447669 | 1.01006457842527 |
| location.rates.West_Asia.Yunnan | 1.00615896686586 | 1.01538051954356 |
| location.rates.West_Asia.Guangdong | 1.00593690847978 | 1.0105070397876 |
| joint | 1.00576622215132 | 1.01417889851427 |
| location.rates.North_America.South_Asia | 1.00535188919861 | 1.01238082607087 |
| location.rates.Oceania.Africa | 1.0049200379241 | 1.01184594104127 |
| location.rates.Africa.West_Asia | 1.00473191836261 | 1.01079638025744 |
| location.indicators.Europe.Africa | 1.00461341941205 | 1.01378195239295 |
| location.rates.Europe.Africa | 1.0045101427484 | 1.01002384647983 |
| location.indicators.Southeast_Asia.Oceania | 1.00438640611976 | 1.00688794235948 |
| location.rates.South_America.Guangdong | 1.00409188160081 | 1.00409751941931 |
| location.indicators.South_America.Oceania | 1.00408141745481 | 1.00649940100873 |
| location.indicators.West_Asia.South_America | 1.00395309678383 | 1.01738070723892 |
| location.indicators.Africa.Oceania | 1.00389964366329 | 1.00583028863756 |
| location.rates.Yunnan.North_America | 1.00376054674956 | 1.01826221707668 |
| location.rates.Europe.South_Asia | 1.00369645079639 | 1.01299946595621 |
| gtr.rates.rateGT | 1.00369195068358 | 1.01887191460547 |
| location.indicators.South_America.Guangdong | 1.00354881869221 | 1.00623552483174 |
| location.rates.South_America.Africa | 1.00336210541974 | 1.00939977774722 |
| age(untitled0) | 1.00326949104772 | 1.01758257732255 |
| tmrca(untitled0) | 1.00326949104739 | 1.01758257732221 |
| location.indicators.West_Asia.Guangdong | 1.00320006870632 | 1.00865434145141 |
| location.rates.South_America.West_Asia | 1.00314913171564 | 1.00733965058522 |
| likelihood | 1.0030919328894 | 1.01553316721397 |
| location.rates.Europe.South_America | 1.00308166859139 | 1.0042455416201 |
| location.rates.Southeast_Asia.Yunnan | 1.00292284933739 | 1.00896141938146 |
| location.rates.Guangdong.Oceania | 1.0028601137311 | 1.00927808649714 |
| location.rates.Guangdong.Europe | 1.00283966604307 | 1.00303128918424 |
| location.rates.Guangdong.North_America | 1.002811029 | 1.00648864909146 |
| location.rates.Southeast_Asia.South_Asia | 1.0027582892932 | 1.00657384200224 |
| location.indicators.Yunnan.Oceania | 1.00273059543868 | 1.00568733342763 |
| location.rates.Guangdong.West_Asia | 1.00272037243078 | 1.00604959040277 |
| location.rates.West_Asia.South_America | 1.00264231046457 | 1.00832916413202 |
| location.indicators.North_America.Yunnan | 1.0026209569128 | 1.0062803411317 |
| location.indicators.Oceania.South_America | 1.00259023063334 | 1.00757340248941 |
| location.indicators.West_Asia.Oceania | 1.00255817254082 | 1.00693871272232 |
| location.indicators.Southeast_Asia.South_Asia | 1.00248220831642 | 1.00513657819459 |
| location.rates.Europe.West_Asia | 1.00243010979877 | 1.00885643557678 |
| location.rates.Guangdong.Yunnan | 1.00239088686633 | 1.00806188354664 |
| location.rates.West_Asia.Africa | 1.00234587409226 | 1.01208241278651 |
| location.indicators.Yunnan.West_Asia | 1.00228324651024 | 1.00466857504182 |
| location.indicators.Southeast_Asia.Europe | 1.00223180610776 | 1.0042478260466 |
| location.clock.rate | 1.00214740080204 | 1.00786826173165 |
| location.meanRate | 1.00214740080204 | 1.00786826173165 |
| location.nonZeroRates | 1.00213888485773 | 1.00287831480389 |
| location.rates.North_America.Yunnan | 1.00213220060685 | 1.00220814498561 |
| alpha | 1.00212887083528 | 1.01193206775158 |
| age(root) | 1.00208338178407 | 1.00869629717431 |
| rootHeight | 1.00208338178397 | 1.00869629717421 |
| location.rates.Yunnan.West_Asia | 1.00208202969556 | 1.00278534307745 |
| location.rates.North_America.Africa | 1.00199676217668 | 1.0026649496435 |
| location.indicators.Africa.Europe | 1.00193761380899 | 1.00303257444117 |
| location.rates.Southeast_Asia.South_America | 1.00192559029534 | 1.00351580033959 |
| location.indicators.West_Asia.South_Asia | 1.00191122894995 | 1.00666177581223 |
| location.rates.Africa.Yunnan | 1.00190100412532 | 1.00546904182045 |
| location.rates.Europe.North_America | 1.00186274379046 | 1.00186769899569 |
| location.rates.West_Asia.Southeast_Asia | 1.00183204271717 | 1.00243193416525 |
| location.rates.Africa.South_America | 1.0017651040006 | 1.00280995112796 |
| gtr.rates.rateCG | 1.0016928983076 | 1.00698488741092 |
| location.rates.South_Asia.Guangdong | 1.00151757433411 | 1.00758999688022 |
| location.rates.Oceania.Guangdong | 1.0014635062516 | 1.00217105679808 |
| location.indicators.Yunnan.Africa | 1.00142222908472 | 1.00368367086194 |
| location.rates.West_Asia.Europe | 1.00133943363452 | 1.00269940154273 |
| location.indicators.Africa.West_Asia | 1.00125526152941 | 1.00442009119151 |
| location.rates.North_America.Oceania | 1.00122859775684 | 1.00623040960171 |
| location.indicators.Guangdong.West_Asia | 1.00121570830498 | 1.00320847537999 |
| location.indicators.Europe.Southeast_Asia | 1.00121060576282 | 1.00333924255158 |
| default.coefficientOfVariation | 1.00119796716306 | 1.00720065189993 |
| location.indicators.North_America.South_America | 1.00118691762089 | 1.00607560197555 |
| location.rates.Africa.South_Asia | 1.00118632346987 | 1.00458398793448 |
| c_allTransitions[1] | 1.00107078672081 | 1.00121587598013 |
| location.rates.South_Asia.Oceania | 1.00106925246582 | 1.00387331920587 |
| location.rates.South_Asia.Southeast_Asia | 1.00104012963997 | 1.00115171836832 |
| location.rates.Yunnan.South_Asia | 1.00102142066349 | 1.00154986902949 |
| location.rates.South_America.Oceania | 1.00101830603654 | 1.00494976840437 |
| location.indicators.Southeast_Asia.Africa | 1.00090931339005 | 1.00233293103071 |
| default.treeLikelihood | 1.00090598823884 | 1.00440412543138 |
| location.indicators.Yunnan.Europe | 1.00090418077515 | 1.00195901598412 |
| location.indicators.Yunnan.Guangdong | 1.00090418077515 | 1.00195901598412 |
| location.indicators.South_America.Africa | 1.00088777410257 | 1.00205640282542 |
| location.indicators.North_America.Europe | 1.00087310282032 | 1.0026462462335 |
| tmrca(untitled1) | 1.00082076079001 | 1.00442995909569 |
| age(untitled1) | 1.00082076077269 | 1.00442995907834 |
| location.rates.Guangdong.Africa | 1.0007929549507 | 1.00315673940649 |
| location.rates.Yunnan.South_America | 1.00079237582625 | 1.00462538551585 |
| location.indicators.Oceania.West_Asia | 1.00075618314444 | 1.00244784209832 |
| location.rates.Oceania.South_America | 1.00071669611143 | 1.00420870747524 |
| location.indicators.South_America.West_Asia | 1.00071347550642 | 1.00146582863191 |
| location.indicators.Oceania.Yunnan | 1.00069431971978 | 1.00245757374942 |
| location.rates.Africa.Oceania | 1.00068663071159 | 1.00263368248415 |
| location.indicators.Guangdong.Africa | 1.00068177612471 | 1.00278749010274 |
| location.indicators.Europe.Yunnan | 1.00064276303462 | 1.00202621005206 |
| location.rates.Europe.Oceania | 1.00058810183702 | 1.00085285323462 |
| location.indicators.Yunnan.South_America | 1.00055398213841 | 1.00145599638074 |
| location.indicators.North_America.Africa | 1.00053145616116 | 1.00227125809952 |
| location.rates.Southeast_Asia.Africa | 1.00052119600698 | 1.00060633898954 |
| location.indicators.North_America.Southeast_Asia | 1.00049811183115 | 1.00167984408342 |
| location.indicators.West_Asia.Europe | 1.00044865322343 | 1.00159782367911 |
| location.indicators.Yunnan.North_America | 1.0004351692837 | 1.00118203127458 |
| c_location.count[1] | 1.00041240775756 | 1.00084166533903 |
| location.rates.Oceania.Southeast_Asia | 1.00039926116442 | 1.00040637006849 |
| location.indicators.Oceania.Africa | 1.00039313875429 | 1.00220905626275 |
| location.indicators.South_Asia.West_Asia | 1.00038921177664 | 1.0012095512614 |
| location.indicators.South_Asia.Guangdong | 1.00034428216912 | 1.00069010369519 |
| default.ucld.stdev | 1.00033771656969 | 1.00134046566948 |
| location.indicators.Oceania.Europe | 1.00033192942606 | 1.00140022316796 |
| location.rates.Yunnan.Europe | 1.00032673843138 | 1.00224613617404 |
| location.rates.South_America.South_Asia | 1.00032615587698 | 1.00032773839494 |
| location.rates.Oceania.Europe | 1.00032371556074 | 1.00302141816351 |
| location.rates.Yunnan.Southeast_Asia | 1.00030634065769 | 1.00186603040461 |
| location.indicators.South_Asia.South_America | 1.00026718875566 | 1.00076245224235 |
| location.rates.South_America.North_America | 1.00023079196185 | 1.00183173448318 |
| location.rates.Southeast_Asia.Guangdong | 1.00021106041207 | 1.00079394259498 |
| location.rates.North_America.Southeast_Asia | 1.00019650617644 | 1.00246749450595 |
| location.rates.Africa.Guangdong | 1.00018989508618 | 1.00073217693087 |
| location.indicators.Guangdong.Yunnan | 1.000181971 | 1.00080446056769 |
| location.rates.Southeast_Asia.Europe | 1.0001731960842 | 1.00101904993026 |
| location.rates.South_Asia.Europe | 1.00016131433189 | 1.00077219203246 |
| location.rates.Southeast_Asia.Oceania | 1.00015568855824 | 1.00209807801378 |
| gtr.rates.rateCT | 1.00015009977642 | 1.00220455683007 |
| location.indicators.Europe.North_America | 1.00015001974711 | 1.00099577833992 |
| location.rates.South_Asia.Yunnan | 1.00014610167107 | 1.00031450175619 |
| location.rates.South_America.Europe | 1.00013812154819 | 1.00146324061232 |
| location.rates.Yunnan.Guangdong | 1.0001081650791 | 1.00086599307502 |
| location.indicators.Oceania.South_Asia | 1.00010268539064 | 1.0009900661867 |
| location.indicators.North_America.Guangdong | 1.00009627616269 | 1.00067047543198 |
| location.rates.Southeast_Asia.North_America | 1.00004440300345 | 1.00040611791813 |
| location.rates.South_America.Southeast_Asia | 1.00001245658099 | 1.0015369391146 |
| location.rates.Oceania.West_Asia | 1.00001230224739 | 1.00012644871654 |
| location.rates.Oceania.South_Asia | 1.00000234503292 | 1.0001060796846 |
| location.rates.North_America.Guangdong | 0.999994704236156 | 1.0000705127668 |
| location.indicators.Southeast_Asia.South_America | 0.99999219692123 | 1.00036901439444 |
| location.rates.Guangdong.South_America | 0.999991286300031 | 1.00007801484978 |
| location.indicators.South_America.Europe | 0.999985836339813 | 1.00010703642187 |
| location.rates.West_Asia.Oceania | 0.999975022044167 | 1.00090748832932 |
| location.indicators.Guangdong.Oceania | 0.99995885218453 | 1.00044534613021 |
| location.indicators.Europe.South_Asia | 0.999958116715362 | 1.00057422402392 |
| location.rates.Africa.North_America | 0.999951437274055 | 1.00024991007437 |
| location.rates.South_Asia.Africa | 0.999943887300798 | 1.001146664 |
| location.rates.South_Asia.South_America | 0.999940332867096 | 1.00106412320177 |
| gtr.rates.rateAC | 0.999931621694104 | 1.00095692306908 |
| location.indicators.South_Asia.North_America | 0.999874624338526 | 1.00029803377199 |
| location.rates.North_America.South_America | 0.999859900208482 | 0.999988192877029 |
| location.indicators.Yunnan.South_Asia | 0.999853609532919 | 1.00010092269049 |
| location.indicators.South_Asia.Yunnan | 0.999845805086271 | 0.999940532873587 |
| location.rates.West_Asia.North_America | 0.999843681593813 | 0.999969290533677 |
| location.indicators.Europe.West_Asia | 0.999834369685438 | 1.00017208144461 |
| pInv | 0.99983051959152 | 0.999844887028207 |
| location.rates.Guangdong.South_Asia | 0.999819333430286 | 1.00031382985641 |
| location.rates.Yunnan.Oceania | 0.999802635696957 | 1.00049707697926 |
| location.rates.North_America.West_Asia | 0.999796270894579 | 1.00024012642663 |
| location.rates.Africa.Southeast_Asia | 0.999793429426902 | 0.999819754969234 |
| location.indicators.Africa.Southeast_Asia | 0.999790445443447 | 0.999872362181309 |
| location.indicators.Africa.South_Asia | 0.999785077438535 | 0.999865642260472 |
| location.rates.West_Asia.South_Asia | 0.999785041219293 | 1.00040628568073 |
| location.indicators.Europe.South_America | 0.999777916774059 | 1.0000243644807 |
| location.rates.Guangdong.Southeast_Asia | 0.999777630909946 | 0.999783467071517 |
| location.rates.Oceania.Yunnan | 0.999769228469057 | 1.00030924179517 |
| location.rates.Oceania.North_America | 0.999765672254313 | 0.999812200579294 |
| location.rates.South_America.Yunnan | 0.999748410879873 | 0.999821480336121 |
| location.indicators.Europe.Guangdong | 0.999744722527305 | 0.999887234553873 |
| location.indicators.Europe.Oceania | 0.999727273027607 | 0.999859083964289 |
| location.indicators.Guangdong.South_America | 0.999719055612127 | 0.99994962367123 |
| location.indicators.Oceania.Southeast_Asia | 0.999716557289873 | 0.999841319178864 |
| location.indicators.Oceania.Guangdong | 0.999715590626533 | 0.999839695898828 |
| location.indicators.Oceania.North_America | 0.999714641220784 | 0.999838098018427 |
| location.indicators.West_Asia.Southeast_Asia | 0.999706809285669 | 0.999824773082662 |
| location.rates.Europe.Yunnan | 0.999694444383748 | 0.999697341222419 |
| gtr.rates.rateAG | 0.999686264487378 | 0.999687154617564 |
| location.indicators.Africa.Yunnan | 0.999684731265923 | 0.999708632807527 |
| location.indicators.West_Asia.Africa | 0.999678530907593 | 0.999773882756918 |
| location.indicators.Africa.South_America | 0.999673738651662 | 0.9998424437136 |
| location.rates.South_Asia.West_Asia | 0.999670875703136 | 0.999835900032559 |
| location.rates.Europe.Guangdong | 0.999669891874334 | 0.999764434557223 |
| location.indicators.Guangdong.North_America | 0.999664492554659 | 0.999724389503378 |
| location.indicators.South_America.Southeast_Asia | 0.999664248505538 | 0.999683286169113 |
| location.indicators.Guangdong.Europe | 0.99966262224441 | 0.999700668511093 |
| location.indicators.Africa.North_America | 0.999658543458838 | 0.999713455852047 |
| location.indicators.Guangdong.Southeast_Asia | 0.999658353087527 | 0.999693973164657 |
| location.indicators.North_America.Oceania | 0.999655862239754 | 0.999689983204136 |
| location.indicators.West_Asia.North_America | 0.999652191825267 | 0.999701181196598 |
| location.rates.South_Asia.North_America | 0.999652031584278 | 0.99970111322101 |
| location.indicators.West_Asia.Yunnan | 0.999648478036665 | 0.999677679996225 |
| location.indicators.Southeast_Asia.North_America | 0.999647725707383 | 0.999676376276947 |
| location.rates.Europe.Southeast_Asia | 0.999636636064036 | 0.999653191188269 |
| location.rates.Africa.Europe | 0.999635737292782 | 0.99964814150203 |
| location.indicators.Guangdong.South_Asia | 0.999633550046597 | 0.999640290494229 |
| gtr.rates.rateAT | 0.999631464852232 | 0.999631470770869 |
| location.indicators.North_America.South_Asia | NA | NA |
| location.indicators.South_America.South_Asia | NA | NA |
| location.indicators.Southeast_Asia.West_Asia | NA | NA |
