# Supplementary material for: Genomic epidemiology reveals the origins and transmission dynamics of chikungunya virus in China
Source: Infect Dis Poverty. 2026 Jun 4;15:64. doi: 10.1186/s40249-026-01465-2 (PMC13234983; doi:10.1186/s40249-026-01465-2)
Supplement: Supplementary file 4 — Supplementary material 4: Table S4. GenBank, GISAID, and GenBase accession numbers of CHIKV sequences used in this study and amino acid variation at positively selected sites in E1 and E2 proteins (imported cases). [file 40249_2026_1465_MOESM4_ESM.docx]

**Table S2** PSRF values for parameters estimated from Bayesian MCMC analyses of the Asian lineage dataset.

| Parameter | PSRF | Upper_CI |
| --- | --- | --- |
| default.covariance | 1.02297581366855 | 1.09268961134743 |
| default.coefficientOfVariation | 1.01644730676544 | 1.03862843299494 |
| c_location.count[1] | 1.008932791 | 1.01931779933735 |
| location.rates.Oceania.North_America | 1.00785364192673 | 1.01051492484597 |
| default.treeLikelihood | 1.00711953293359 | 1.02890552199432 |
| location.rates.South_America.Yunnan | 1.00578372691004 | 1.01585899537926 |
| location.indicators.Yunnan.South_Asia | 1.0057172154666 | 1.0239782090471 |
| location.rates.North_America.Oceania | 1.00539640148206 | 1.00639990492362 |
| tmrca(untitled0) | 1.00535478226177 | 1.02786528469534 |
| age(untitled0) | 1.00535478226106 | 1.02786528469462 |
| location.rates.South_Asia.Oceania | 1.0049279423967 | 1.00497300627227 |
| location.rates.Southeast_Asia.Yunnan | 1.00490022457378 | 1.01037230338428 |
| likelihood | 1.00449878676311 | 1.01934533201041 |
| location.rates.South_Asia.South_America | 1.00438219334597 | 1.00599324263551 |
| location.rates.South_America.North_America | 1.00427972528559 | 1.01632698646095 |
| c_allTransitions[1] | 1.00385791052057 | 1.00979298603744 |
| location.rates.Oceania.South_Asia | 1.00383273735012 | 1.00623390499497 |
| location.rates.Southeast_Asia.South_America | 1.00362955997562 | 1.00683392063681 |
| location.rates.Yunnan.North_America | 1.00325128175466 | 1.00562189532366 |
| location.indicators.Southeast_Asia.North_America | 1.00294495549357 | 1.00351940376457 |
| location.rates.North_America.Yunnan | 1.00286651769733 | 1.00286707047394 |
| location.rates.South_America.Oceania | 1.00212900382184 | 1.00518431556707 |
| location.rates.South_America.Southeast_Asia | 1.0018211757217 | 1.00456400820666 |
| gtr.rates.rateAC | 1.00169938366218 | 1.00195413980715 |
| location.indicators.South_America.Yunnan | 1.00139974644841 | 1.00597792950439 |
| location.indicators.South_America.Southeast_Asia | 1.00136380728138 | 1.00639523402701 |
| location.rates.Oceania.South_America | 1.00129949412208 | 1.00210335801106 |
| location.indicators.North_America.Oceania | 1.00128411331539 | 1.00364553190297 |
| location.rates.Southeast_Asia.South_Asia | 1.00125559073836 | 1.0039764498536 |
| location.indicators.South_Asia.Yunnan | 1.00120113277428 | 1.00532403008913 |
| default.meanRate | 1.00117345087143 | 1.00789631379575 |
| location.indicators.Yunnan.Oceania | 1.00101859288337 | 1.00516277253132 |
| location.rates.Yunnan.South_America | 1.00099910012302 | 1.00100462669013 |
| location.rates.South_America.South_Asia | 1.0009920662686 | 1.00612950018388 |
| location.rates.Yunnan.Southeast_Asia | 1.00094264080437 | 1.00492161863925 |
| gtr.rates.rateCT | 1.00087203375529 | 1.00603785632352 |
| location.indicators.South_America.South_Asia | 1.0008504928803 | 1.00415037437255 |
| location.indicators.Oceania.Yunnan | 1.00077255915346 | 1.00503375539188 |
| location.rates.North_America.Southeast_Asia | 1.00070461520926 | 1.00077882661502 |
| location.rates.Yunnan.Oceania | 1.00067390604205 | 1.0008563303972 |
| location.indicators.Yunnan.Southeast_Asia | 1.00065013617334 | 1.00412253925916 |
| location.indicators.South_Asia.Southeast_Asia | 1.00056435881235 | 1.00492093784081 |
| location.rates.North_America.South_America | 1.00048785383852 | 1.00456116015692 |
| location.indicators.Yunnan.North_America | 1.00047045415498 | 1.00388976299194 |
| joint | 1.00044165421675 | 1.00403793019726 |
| location.rates.South_Asia.North_America | 1.0004350857645 | 1.00139026316698 |
| location.indicators.North_America.Yunnan | 1.00042134264441 | 1.00183386422108 |
| location.rates.Oceania.Yunnan | 1.0004202094761 | 1.00224786156512 |
| location.indicators.Southeast_Asia.Yunnan | 1.00041009572376 | 1.00283549585814 |
| gtr.rates.rateAG | 1.00035210652633 | 1.0039661 |
| default.ucld.stdev | 1.00030134424003 | 1.00336035258586 |
| default.ucld.mean | 1.00029715076364 | 1.00061810413018 |
| location.indicators.South_Asia.South_America | 1.00024540397807 | 1.00183738300268 |
| location.rates.South_Asia.Southeast_Asia | 1.00021962165048 | 1.00193368962333 |
| location.indicators.Southeast_Asia.South_America | 1.00020417872986 | 1.00222136636301 |
| location.indicators.Yunnan.South_America | 1.00017782749862 | 1.00216304019548 |
| location.indicators.Southeast_Asia.South_Asia | 1.00016927128249 | 1.00294206492907 |
| location.indicators.North_America.Southeast_Asia | 1.00006832861704 | 1.00081339387517 |
| gtr.rates.rateCG | 1.00005749525712 | 1.00016295663344 |
| location.rates.Yunnan.South_Asia | 1.00002087146115 | 1.0009293605193 |
| location.clock.rate | 0.999961512137065 | 1.00098994799419 |
| location.meanRate | 0.999961512137065 | 1.00098994799419 |
| location.rates.Southeast_Asia.North_America | 0.999857666788802 | 1.0004290414887 |
| location.indicators.South_America.Oceania | 0.999829515334782 | 1.00096910180009 |
| location.rates.Southeast_Asia.Oceania | 0.999813469072166 | 1.00029466167106 |
| location.indicators.North_America.South_Asia | 0.999797756592297 | 1.00026680629065 |
| treeLength | 0.999748392926286 | 1.00081031812673 |
| location.nonZeroRates | 0.99972306325198 | 0.999969552924296 |
| location.indicators.South_Asia.North_America | 0.999722551742653 | 1.00041603740444 |
| gtr.rates.rateAT | 0.999700214151915 | 1.00038909816327 |
| location.rates.South_Asia.Yunnan | 0.999630329116592 | 0.999733060903172 |
| location.rates.North_America.South_Asia | 0.999629002284264 | 0.999895381864598 |
| gtr.rates.rateGT | 0.999627830110915 | 1.00009060558039 |
| location.indicators.Oceania.North_America | 0.999584255324717 | 0.999954743394758 |
| location.indicators.South_America.North_America | 0.999575780132409 | 0.999935952091479 |
| location.rates.Oceania.Southeast_Asia | 0.999564629420708 | 0.999593908888737 |
| prior | 0.999559246331858 | 0.999980515060648 |
| location.indicators.South_Asia.Oceania | 0.999555036805475 | 0.999769270636151 |
| location.indicators.Oceania.South_America | 0.999526598015204 | 0.999713318206805 |
| alpha | 0.999519970103526 | 0.999712233467481 |
| constant.popSize | 0.999477185477656 | 0.999523933312122 |
| coalescent | 0.999470956824254 | 0.999573327089701 |
| age(root) | 0.99946786424045 | 0.999495413276277 |
| rootHeight | 0.99946786424044 | 0.999495413276267 |
| location.indicators.Oceania.South_Asia | 0.999456223711604 | 0.999492015615848 |
| location.indicators.Oceania.Southeast_Asia | 0.999448604130457 | 0.999458685821757 |
| location.indicators.Southeast_Asia.Oceania | NA | NA |
