# Supplementary material for: Genomic epidemiology reveals the origins and transmission dynamics of chikungunya virus in China
Source: Infect Dis Poverty. 2026 Jun 4;15:64. doi: 10.1186/s40249-026-01465-2 (PMC13234983; doi:10.1186/s40249-026-01465-2)
Supplement: Supplementary file 5 — Supplementary material 5: Table S5. GenBank and GenBase accession numbers of CHIKV sequences used in this study and amino acid variation at positively selected sites in E1 and E2 proteins (local transmission cases). [file 40249_2026_1465_MOESM5_ESM.docx]

**Table S3.** Summary of reported chikungunya cases in China.

| **Reporting Region** | **Year** | **Number of Cases** | **Case Classification** | **Country of Origin (Imported Cases)** | **Reference(s)** |
| --- | --- | --- | --- | --- | --- |
| Yunnan | 1987 | 1 | local |  | [1] |
| Hong Kong | 2006 | 3 | Imported | Mauritius(1), Unknown(2) | [2,3] |
| Taiwan | 2006 | 1 | Imported | Singapore | [4] |
| Taiwan | 2007 | 3 | Imported | Indonesia | [4] |
| Guangdong | 2008 | 5 | Imported | Sri Lanka(2), Malaysia(3) | [5,6] |
| Zhejiang | 2008 | 1 | Imported | Malaysia | [7] |
| Hong Kong | 2008 | 3 | Imported | Unknown | [3] |
| Taiwan | 2008 | 9 | Imported | Indonesia(4), Malaysia(3), Bangladesh(1), India(1) | [4] |
| Guangdong | 2009 | 1 | Imported | Unknown | [6] |
| Taiwan | 2009 | 9 | Imported | Indonesia(4), Malaysia(2),Thailand(2), Singapore(1) | [4,8] |
| Guangdong | 2010 | 309 | local |  | [9,10] |
| Guangdong | 2010 | 5 | Imported | Ethiopia(1), India(1),Unknown(3) | [11] |
| Taiwan | 2010 | 13 | Imported | Indonesia(12), Malaysia(1) | [4,8] |
| Guangdong | 2011 | 1 | Imported | Unknown | [6] |
| Shandong | 2011 | 1 | Imported | Congo | [12] |
| Taiwan | 2011 | 2 | Imported | Philippines(1), Myanmar(1) | [4,8] |
| Guangdong | 2012 | 2 | Imported | Indonesia(1), Thailand(1) | [13,14] |
| Fujian | 2012 | 1 | Imported | Philippines | [15] |
| Zhejiang | 2012 | 2 | Imported | Philippines(1), Unknown(1) | [16,17] |
| Taiwan | 2012 | 5 | Imported | 1Indonesia(1), Philippines(3), Cambodia(1) | [4,8] |
| Guangdong | 2013 | 3 | Imported | Indonesia(2), Malaysia(1) | [18,19] |
| Fujian | 2013 | 1 | Imported | Unknown | [20] |
| Taiwan | 2013 | 29 | Imported | Indonesia(17), Philippines(8), Thailand(2), Singapore(2) | [4,8] |
| Shandong | 2014 | 2 | Imported | Thailand | [21] |
| Zhejiang | 2014 | 1 | Imported | Unknown | [7] |
| Taiwan | 2014 | 7 | Imported | Indonesia(5), Philippines(1), Guatemala(1) | [4,8] |
| Taiwan | 2015 | 4 | Imported | Unknown | [8] |
| Guangdong | 2016 | 2 | Imported | Unknown | [22] |
| Zhejiang | 2016 | 2 | Imported | Unknown | [7] |
| Hong Kong | 2016 | 2 | Imported | India | [23] |
| Taiwan | 2016 | 14 | Imported | Unknown | [8] |
| Zhejiang | 2017 | 3 | local |  | [24] |
| Beijing | 2017 | 1 | Imported | Bangladesh | [25] |
| Henan | 2017 | 1 | Imported | Sri Lanka | [26] |
| Zhejiang | 2017 | 6 | Imported | Bangladesh(4), Unknown(2) | [7,24] |
| Taiwan | 2017 | 11 | Imported | Unknown | [8] |
| Guangdong | 2017 | 23 | Imported | Bangladesh(12), Pakistan(3), Ethiopia(1), Unknown(7) | [22,27,28,29] |
| Guangdong | 2018 | 6 | Imported | India(2), Philippines(1), Kenya(1), Cambodia(1), Congo(1) | [30] |
| Fujian | 2018 | 1 | Imported | Philippines | [31] |
| Chongqing | 2018 | 1 | Imported | Thailand | [32] |
| Zhejiang | 2018 | 1 | Imported | Unknown | [7] |
| Taiwan | 2018 | 7 | Imported | Unknown | [8] |
| Yunnan | 2019 | 229 | local |  | [33-36] |
| Taiwan | 2019 | 21 | local |  | [8,37] |
| Yunnan | 2019 | 54 | Imported | Myanmar(40), Thailand(4), Cambodia(1), Unknown(9) | [35,38]; MN402883-MN402892, PV022110-PV022127 |
| Zhejiang | 2019 | 12 | Imported | Myanmar(1), Thailand(2), Unknown(9) | [7,39,40] |
| Guangdong | 2019 | 22 | Imported | Thailand(8), Myanmar(5), India(2), Maldives(3), Malaysia(1), Cambodia(1), Ethiopia(1), Philippines(1) | [29,30,41] |
| Beijing | 2019 | 1 | Imported | Myanmar | [25] |
| Guizhou | 2019 | 1 | Imported | Myanmar | [42] |
| Henan | 2019 | 1 | Imported | Myanmar | [43] |
| Liaoning | 2019 | 1 | Imported | Myanmar | [44] |
| Shandong | 2019 | 1 | Imported | Myanmar | [45] |
| Tianjin | 2019 | 1 | Imported | Myanmar | [46] |
| Taiwan | 2019 | 86 | Imported | Myanmar(53), Unknown(33) | [39] |
| Yunnan | 2020 | 3 | Imported | Myanmar(2), Cambodia(1) | PV022121, PV022122, PV022123 |
| Zhejiang | 2020 | 2 | Imported | Unknown | [7] |
| Guangdong | 2023 | 2 | Imported | Philippines(1), India(1) | OR715104, OR785139 |
| Sichuan | 2023 | 1 | Imported | Unknown | [47] |
| Guangdong | 2024 | 4 | Imported | Indonesia(5), Timor-Leste(1),Unknown(2) | PP554682, PP798381, C_AA119977, C_AA119975 |
| Zhejiang | 2024 | 1 | Imported | Indonesia | [48] |
| Shanghai | 2024 | 1 | Imported | Maldives | [49] |
| Guangdong | 2025 | 25,335 | local |  | [50,51]; https://cdcp.gd.gov.cn/ywdt/zdzt/yfjkkyr/yqxx/content/post_4800622.html |
| Guangxi | 2025 | 50 | local |  | [52] |
| Guangdong | 2025 | 23 | Imported | Unknown | C_AA119980, C_AA131416, C_AA131424, C_AA131415, C_AA131423, C_AA131414, C_AA131417, C_AA131418, C_AA131419, C_AA131420, C_AA131426, C_AA131427, C_AA131428, C_AA131421, C_AA131422, C_AA131425, C_AA119976, C_AA119979, C_AA119981, C_AA119978, C_AA119982, C_AA119984, C_AA119983 |
| Beijing | 2025 | 1 | Imported | Ethiopia | [53] |
| Jiangsu | 2025 | 2 | Imported | Indonesia(1), Unknown(1) | PX425075, EPI_ISL_20136536 |
| Shanghai | 2025 | 1 | Imported | Unknown | PV805110 |
| Ningxia | 2025 | 1 | Imported | Unknown | C_AA130858 |
| Yunnan | 2025 | 1 | Imported | Myanmar | PX921692 |
| Hainan | 2025 | 1 | Imported | Germany | [54] |

1. Shi H, Zhang H, Zi D, Mi Z, Li Z, Liu L, et al. Chikungunya virus was first isolated from the patient in Yunnan Province. Chin J Zoonoses. 1990;6(1):2–4. (In Chinese).
2. Lee N, Wong CK, Lam WY, Wong A, Lim W, Lam CW, et al. Chikungunya fever, Hong Kong. Emerg Infect Dis. 2006;12:1790–2.
3. Ma SK, Wong WC, Leung CW, Lai ST, Lo YC, Wong KH, et al. Review of vector-borne diseases in Hong Kong. Travel Med Infect Dis. 2011;9:95–105.
4. Yang CF, Su CL, Hsu TC, Chang SF, Lin CC, Huang JC, et al. Imported Chikungunya virus strains, Taiwan, 2006–2014. Emerg Infect Dis. 2016;22:1981–4.
5. Lin M, Li H, Dai J, Huang L, Huang J, Zheng K, et al. Building impregnable fortress for infectious disease prevention through integrating inspection, laboratory test and rapid cooperative respond at port. Chin J Front Health Quar. 2009;32(5):293–6. (In Chinese).
6. Huang L, Zhang X, Zhang W, Deng J, Lin S, Wu H, et al. Analysis of imported chikungunya fever cases detected at frontier ports of Guangdong Entry-Exit Inspection and Quarantine Bureau. Chin J Vector Biol Control. 2013;24(4):357–60. (In Chinese).
7. Ren J, Ling F, Liu Y, Sun J. Chikungunya in Zhejiang Province, Southeast China. Infect Med (Beijing). 2023;2:315–23.
8. Chou YC, Hsieh CJ, Cheng CA, Wu DC, Wu WC, Lin FH, et al. Epidemiologic characteristics of imported and domestic chikungunya cases in Taiwan: a 13-year retrospective study. Int J Environ Res Public Health. 2020;17:3615.
9. Wu D, Zhang Y, Zhouhui Q, Kou J, Liang W, Zhang H, et al. Chikungunya virus with E1-A226V mutation causing two outbreaks in 2010, Guangdong, China. Virol J. 2013;10:174.
10. Chen B, Chen Q, Li Y, Mu D, Wang Z, Zhu M, et al. Epidemiological characteristics of imported Chikungunya fever cases in China, 2010–2019. Dis Surveill. 2021;36(6):539–43. (In Chinese).
11. Lu X, Li X, Mo Z, Jin F, Wang B, Huang J, et al. Chikungunya emergency in China: microevolution and genetic analysis for a local outbreak. Virus Genes. 2014;48:15–22.
12. Li D, Zheng W. Prevention and control strategies of epidemiological investigation of first case of Chikungunya fever in Zibo. J Qiqihar Med Univ. 2013;34(9):1343–4. (In Chinese).
13. Zhang X, Huang Y, Wang M, Yang F, Cheng J, Wan C, et al. Complete genome sequences of two chikungunya viruses imported into China. Genome Announc. 2018;6(26):e00480-18.
14. Xu S, Ren Y, Sun H, Li Y, Cai J, He X, et al. Molecular genetic analysis of two Chikungunya virus strains isolated in Shenzhen. China Trop Med. 2015;15(5):539–41. (In Chinese).
15. Cai M, Su Y, Wang T, Wu J, Chen W, Du C. Investigation and management of an imported chikungunya outbreak. Strait J Prev Med. 2013;19(6):84–5. (In Chinese).
16. Zhou D, Luo J. Complete genome sequence characteristics of the first identification of Chikungunya virus in Zhejiang province, China. Chin J Front Health Quar. 2018;41(6):391–5. (In Chinese).
17. Sun Y, Yan J, Mao H, Zhang L, Lyu Q, Wu Z, et al. Characterization of the complete genome of chikungunya in Zhejiang, China, using a modified virus discovery method based on cDNA-AFLP. PLoS One. 2013;8:e83014.
18. Shi L, Fu S, Wang L, Li X, Gu D, Liu C, et al. Surveillance of mosquito-borne infectious diseases in febrile travelers entering China via Shenzhen ports, China, 2013. Travel Med Infect Dis. 2016;14:123–30.
19. Li X, Huang J, Su J, Fang S, Zheng K. Analysis of vector-borne disease testing for incoming fever patients at Guangdong border ports in 2013. South China J Prev Med. 2015;41(4):395–6. (In Chinese).
20. Lv W, Li F, Li H, Liu J, Hong S. Research on the prevention and control status and emergency response strategies for dengue fever and chikungunya fever in Quanzhou city. Strait J Prev Med. 2015;21(5):45–6. (In Chinese).
21. Zhang J, Xu H, Xue X, Zhu K, Chen X, Zhang J, et al. Identification of imported cases of dual infection with dengue virus and chikungunya virus. Chin J Virol. 2017;33(2):200–4. (In Chinese).
22. Feng X, Sun W, Birkhead GS, Wang X, Guo Z, Lu J. The surveillance of four mosquito-borne diseases in international travelers arriving at Guangzhou Baiyun International Airport, China, 2016–2017. Travel Med Infect Dis. 2019;32:101513.
23. Ho DTW, Chan DPC, Lam CY, Liang DC, Lee SS, Kam JKM. At the advancing front of Chikungunya fever in Asia: two imported cases in Hong Kong with novel amino acid changes. J Microbiol Immunol Infect. 2018;51:419–21.
24. Pan J, Fang C, Yan J, Yan H, Zhan B, Sun Y, et al. Chikungunya fever outbreak, Zhejiang Province, China, 2017. Emerg Infect Dis. 2019;25:1589–91.
25. Tian L, Ma J, Wu K, Wang Q, Jia L, Chen L. Analysis of epidemiological characteristics of imported Aedes mosquito-borne diseases in Beijing. Acta Parasitol Med Entomol Sin. 2020;27(3):151–7. (In Chinese).
26. Li XL, Li Y, Wang RL, Zhang BF, Su J, Guo DC, et al. Analysis on the epidemiology and etiology characteristics of first imported Chikungunya fever case in Henan Province in 2017. Zhonghua Yu Fang Yi Xue Za Zhi. 2019;53:415–8. (In Chinese).
27. Yang Y, Xu Z, Zheng H, Song J, Wu Y, Tong Z, et al. Genetic and phylogenetic characterization of a chikungunya virus imported into Shenzhen, China. Virol Sin. 2020;35:115–9.
28. Wang Z, Ma X, Xie C, Zhen R, Peng Z, Li Y. Investigation and analysis of an imported Chikungunya fever case in Guangzhou. J Med Pest Control. 2019;35(12):1192–4. (In Chinese).
29. Dai J, Yuan S, Liu X, Lu Y, Tian J, Sun W, et al. Epidemiological investigation of imported infectious disease cases among travellers from Africa to China. Chin J Front Health Quar. 2021;44(4):261–7. (In Chinese).
30. Zhen R, Su W, Liao X, Ma X, Li Y, Feng J, et al. Epidemiological and etiological characteristics of imported chikungunya fever cases, Guangzhou. Dis Surveill. 2021;36(1):48–52. (In Chinese).
31. Yu T, Lin Q, Kan N, You L, Weng Y, Wang J. Isolation, identification and complete gene characterization of the first Chikungunya virus strain in Fujian Province, China. Chin J Virol. 2022;38(6):1339–45. (In Chinese).
32. Wen H, Tuo X, Wang D, Zhou L, Zhang Q, Zhou X, et al. Genomic characterization of the first detected imported chikungunya virus at Chongqing port. Chin J Front Health Quar. 2023;46(4):313–5. (In Chinese).
33. Zhao D, Li H, Zhang H, Shi J, Guo J, Yang L, et al. Dengue virus, chikungunya virus, and Zika virus infections in febrile cases in Gengma county, Yunnan province, in 2019. Dis Surveill. 2024;39(6):711–7. (In Chinese).
34. Zou M, Su C, Li T, Zhang J, Li D, Luan N, et al. Genetic characterization of Chikungunya virus among febrile dengue fever-like patients in Xishuangbanna, Southwestern part of China. Front Cell Infect Microbiol. 2022;12:914289.
35. Yang YF, Li XL, Hu TS, Yin XX, Xu Q, Tian Y, et al. The ecological factors contributing to the chikungunya outbreak in Ruili, a border city in Yunnan Province, China. BMC Infect Dis. 2025;25:1643.
36. Liu LB, Li M, Gao N, Shen JY, Sheng ZY, Fan DY, et al. Epidemiological and clinical characteristics of the chikungunya outbreak in Ruili City, Yunnan Province, China. J Med Virol. 2022;94:499–506.
37. Chen MY, Huang AS, Yang CF, Hsu TC, Wang TC, Su CL, et al. Chikungunya infection: first autochthonous cases in Taiwan. J Formos Med Assoc. 2021;120:1526–30.
38. Chen Y, Zhao X, Sun Y, Zhang M, Zhou J. Sequencing and characterization analysis of a chikungunya virus imported into Yunnan Province. Chin J Zoonoses. 2021;37(3):221–5. (In Chinese).
39. Su L, Lou X, Yan H, Yang Z, Mao H, Yao W, et al. Importation of a novel Indian Ocean lineage carrying E1-K211E and E2-V264A of Chikungunya virus in Zhejiang Province, China, in 2019. Virus Genes. 2023;59:693–702.
40. Ren J, Chen Z, Ling F, Liu Y, Chen E, Shi X, et al. The epidemiology of Aedes-borne arboviral diseases in Zhejiang, Southeast China: a 20 years population-based surveillance study. Front Public Health. 2023;11:1270781.
41. Zheng K, Sun F, Yuan S, Li D, Lin Z, Liang J, et al. Identification of a mixed infection case involving chikungunya virus and Zika virus. South China J Prev Med. 2019;45(5):481–5. (In Chinese).
42. Qiu S, Guo J, Li P, Li P, Du X, Hao R, et al. Source-tracking of the Chinese Chikungunya viruses suggests that Indian subcontinent and Southeast Asia act as major hubs for the recent global spread of Chikungunya virus. Virol J. 2021;18:203.
43. Li X, Jin X, Ma J, Li X, Fu R, Wang H, et al. Detection and management of an imported case of Chikungunya fever in Henan. Dis Surveill. 2020;35(3):202–5. (In Chinese).
44. Gao Y, Hou L, Fu S, Jiang Y, Cheng X, Jia Y, et al. Laboratory detection and confirmation of the first imported case of chikungunya fever in Dalian port. Chin J Front Health Quar. 2021;44(5):309–11. (In Chinese).
45. Zhang Y, Jiang X, Chen D, Lv T, Zhang G, Zhang H, et al. An imported case of Chikungunya fever among members of an outbound touring group: case survey and laboratory test. Chin J Public Health. 2021;37(5):879–82. (In Chinese).
46. Xie T, Lyu LK, Tan ZL, Li L, Lyu J, Li XY. Genotyping on one case with Chikungunya infection introduced into Tianjin in China from Myanmar. Zhonghua Liu Xing Bing Xue Za Zhi. 2020;41(12):2131–4. (In Chinese).
47. Wang Y, Dai Y, Liu H, Feng S, Chen Y, Long L, et al. Analysis of a syndromic surveillance system for infectious diseases of the 31st summer World University Games in Chengdu, China. Front Public Health. 2025;13:1510057.
48. Bao W, Fang W, Shi Y, Hu Y. Investigation and disposal of the first imported case of chikungunya fever in Yongkang city, Zhejiang province. Pract Prev Med. 2025;32(10):1228–31. (In Chinese).
49. Ni X, Shen L, Teng Z, Hu H. Investigation and management of an imported case of chikungunya fever in Jing’an District, Shanghai in 2024. China Trop Med. 2025;25(11):1507–10. (In Chinese).
50. Lin D, Zhang D, Chen S, Zeng X, Feng Q, Li M, et al. Preliminary epidemiological analysis of the 2025 Chikungunya outbreak in Guangdong Province. J Trop Med. 2025;25(12):1693–7. (In Chinese).
51. Zhang J, Cai J, Liu C, Dos Santos Costa MT, Ren T, Chen L. The epidemic pattern of a chikungunya outbreak in China exhibits a three-stage migratory trend. J Infect. 2025;91:106646.
52. Liang Y, Xie Z, Li Y, Li S, Qin Y. Epidemiological and clinical characteristics of 50 patients with chikungunya fever: an analytic study. Guangxi Med J. 2025;47(10):1450–3. (In Chinese).
53. Tian R, Zhao J, Li Y, Jiao Y, Bao K, Pan P, et al. Case report on the identification of whole genome sequence of chikungunya virus using probe-capture metagenomics sequencing from throat swab sample. Dis Surveill. 2025;40(10):1349–53. (In Chinese).
54. Shan Y, Chen Y, Huang J, Wu B. The first imported case of chikungunya fever reported in Haikou City, Hainan Province. China Trop Med. 2025;25(12):1637–40. (In Chinese).
