# Supplementary material for: Genomic epidemiology reveals the origins and transmission dynamics of chikungunya virus in China
Source: Infect Dis Poverty. 2026 Jun 4;15:64. doi: 10.1186/s40249-026-01465-2 (PMC13234983; doi:10.1186/s40249-026-01465-2)
Supplement: Supplementary file 6 — Supplementary material 6: Table S6. Annual number of reported CHIKV cases and available sequences in China, 1987–2025. [file 40249_2026_1465_MOESM6_ESM.docx]

**Table S4.** GenBank, GISAID, and GenBase accession numbers of CHIKV sequences used in this study and amino acid variation at positively selected sites in E1 and E2 proteins (imported cases).

| **Accession No.** | **Collection Date** | **Countries of Entry of Patients** | **Province of Importation (China)** | **Genotype** | **Length (nt)** | **Genomic region** | **Amino acid positions under positive selection** | | | | | | |
| --- | --- | --- | --- | --- | --- | --- | --- | --- | --- | --- | --- | --- | --- |
|  |  |  |  |  |  |  | **E2** | | | | **E1** | | |
|  |  |  |  |  |  |  | **60** | **210** | **211** | **264** | **98** | **211** | **226** |
| DQ489787 | 2006 | Mauritius | Hong Kong | Indian Ocean | 1043 | E1 | - | - | - | - | A | K | V |
| GU199351 | 2008 | Sri_Lanka | Guangdong | Indian Ocean | 11,793 | NSP1-E1 | D | L | T | V | A | K | A |
| GU199350 | 2008 | Sri_Lanka | Guangdong | Indian Ocean | 11,687 | NSP1-E1 | D | L | T | V | A | K | A |
| GU199353 | 2008 | Malaysia | Guangdong | Indian Ocean | 11,687 | NSP1-E1 | D | L | T | V | A | K | V |
| GU199352 | 2008 | Malaysia | Guangdong | Indian Ocean | 11,677 | NSP1-E1 | D | L | T | V | A | K | V |
| MH670649 | 2009 | Malaysia | Guangdong | Asian | 11,970 | NSP1-E1 | D | L | T | V | T | E | A |
| HM067743 | 2009 | Singapore | Taiwan | Indian Ocean | 3747 | C-E1 | D | L | T | V | A | K | V |
| HM067744 | 2009 | Thailand | Taiwan | Indian Ocean | 3747 | C-E1 | D | L | T | V | A | K | V |
| JQ067624 | 2010 | unknown | Guangdong | Central African | 11,724 | NSP1-E1 | D | L | T | V | A | K | A |
| MG664850 | 2010 | India | Guangdong | Indian Ocean | 11,844 | NSP1-E1 | D | L | T | A | A | E | A |
| JQ065891 | 2010 | unknown | Guangdong | Indian Ocean | 11,687 | NSP1-E1 | D | L | T | A | A | E | A |
| JQ065890 | 2010 | unknown | Guangdong | Indian Ocean | 11,684 | NSP1-E1 | D | L | T | A | A | E | A |
| JQ065892 | 2010 | unknown | Guangdong | Indian Ocean | 11,153 | NSP1-E1 | D | L | T | A | A | E | A |
| KF318729 | 2012 | unknown | Zhejiang | Asian | 12,017 | NSP1-E1 | D | L | T | V | T | E | A |
| MG664851 | 2012 | Indonesia | Guangdong | Asian | 12,000 | NSP1-E1 | D | L | T | V | T | E | A |
| KC488650 | 2012 | Philippines | Zhejiang | Asian | 11,889 | NSP1-E1 | D | L | T | V | T | E | A |
| MF499120 | 2016 | India | Hong Kong | Indian Ocean | 11,733 | NSP1-E1 | D | L | T | A | A | E | A |
| MF503628 | 2016 | India | Hong Kong | Indian Ocean | 11,656 | NSP1-E1 | D | L | T | A | A | E | A |
| MH349097 | 2017 | unknown | Guangdong | Indian Ocean | 11,783 | NSP1-E1 | D | L | T | A | A | E | A |
| MG912993 | 2017 | Bangladesh | Zhejiang | Indian Ocean | 11,765 | NSP1-E1 | D | L | T | A | A | E | A |
| OR037305 | 2017 | unknown | Guangdong | Indian Ocean | 11,756 | NSP1-E1 | D | L | T | A | A | E | A |
| MG925665 | 2017 | unknown | Henan | Indian Ocean | 11,733 | NSP1-E1 | D | L | T | A | A | E | A |
| EPI_ISL_18131740 | 2017 | unknown | Guangdong | Indian Ocean | 11,538 | NSP1-E1 | D | L | T | A | A | E | A |
| OR037306 | 2018 | unknown | Guangdong | Central African | 11,730 | NSP1-E1 | D | L | I | V | A | K | V |
| OR037308 | 2019 | unknown | Guangdong | Indian Ocean | 11,816 | NSP1-E1 | D | L | T | A | A | E | A |
| OR037307 | 2019 | unknown | Guangdong | Indian Ocean | 11,814 | NSP1-E1 | D | L | T | A | A | E | A |
| OR037309 | 2019 | unknown | Guangdong | Indian Ocean | 11,814 | NSP1-E1 | D | L | T | A | A | E | A |
| MN402883 | 2019 | Thailand | Yunnan | Indian Ocean | 11,793 | NSP1-E1 | D | L | T | A | A | E | A |
| MN402884 | 2019 | Myanmar | Yunnan | Indian Ocean | 11,793 | NSP1-E1 | D | L | T | A | A | E | A |
| MN402885 | 2019 | Myanmar | Yunnan | Indian Ocean | 11,793 | NSP1-E1 | D | L | T | A | A | E | A |
| MN402886 | 2019 | Myanmar | Yunnan | Indian Ocean | 11,793 | NSP1-E1 | D | L | T | A | A | E | A |
| MN402887 | 2019 | Myanmar | Yunnan | Indian Ocean | 11,793 | NSP1-E1 | D | L | T | A | A | E | A |
| MN402888 | 2019 | Myanmar | Yunnan | Indian Ocean | 11,793 | NSP1-E1 | D | L | T | A | A | E | A |
| MN402889 | 2019 | Myanmar | Yunnan | Indian Ocean | 11,793 | NSP1-E1 | D | L | T | A | A | E | A |
| MN402890 | 2019 | Myanmar | Yunnan | Indian Ocean | 11,793 | NSP1-E1 | D | L | T | A | A | E | A |
| MN402891 | 2019 | Myanmar | Yunnan | Indian Ocean | 11,793 | NSP1-E1 | D | L | T | A | A | E | A |
| MN402892 | 2019 | Myanmar | Yunnan | Indian Ocean | 11,793 | NSP1-E1 | D | L | T | A | A | E | A |
| OL840905 | 2019 | Thailand | Zhejiang | Indian Ocean | 11,786 | NSP1-E1 | D | L | T | A | A | E | A |
| OL840906 | 2019 | Thailand | Zhejiang | Indian Ocean | 11,786 | NSP1-E1 | D | L | T | A | A | E | A |
| MT668625 | 2019 | Myanmar | Tianjin | Indian Ocean | 11,727 | NSP1-E1 | D | L | T | A | A | E | A |
| MN756625 | 2019 | Myanmar | Guizhou | Indian Ocean | 11,511 | NSP1-E1 | D | L | T | A | A | E | A |
| OL840907 | 2019 | Myanmar | Zhejiang | Indian Ocean | 11,436 | NSP1-E1 | D | L | T | A | A | E | A |
| PV022110 | 2019 | Myanmar | Yunnan | Indian Ocean | 11,237 | NSP1-E1 | D | L | T | A | A | E | A |
| PV022111 | 2019 | Myanmar | Yunnan | Indian Ocean | 11,237 | NSP1-E1 | D | L | T | A | A | E | A |
| PV022112 | 2019 | Myanmar | Yunnan | Indian Ocean | 11,237 | NSP1-E1 | D | L | T | A | A | E | A |
| PV022113 | 2019 | Myanmar | Yunnan | Indian Ocean | 11,237 | NSP1-E1 | D | L | T | A | A | E | A |
| PV022114 | 2019 | Myanmar | Yunnan | Indian Ocean | 11,237 | NSP1-E1 | D | L | T | A | A | E | A |
| PV022115 | 2019 | Myanmar | Yunnan | Indian Ocean | 11,237 | NSP1-E1 | D | L | T | A | A | E | A |
| PV022116 | 2019 | Myanmar | Yunnan | Indian Ocean | 11,237 | NSP1-E1 | D | L | T | A | A | E | A |
| PV022117 | 2019 | Myanmar | Yunnan | Indian Ocean | 11,237 | NSP1-E1 | D | L | T | A | A | E | A |
| PV022118 | 2019 | Myanmar | Yunnan | Indian Ocean | 11,237 | NSP1-E1 | D | L | T | A | A | E | A |
| PV022119 | 2019 | Thailand | Yunnan | Indian Ocean | 11,237 | NSP1-E1 | D | L | T | A | A | E | A |
| PV022120 | 2019 | Cambodia | Yunnan | Indian Ocean | 11,237 | NSP1-E1 | D | L | T | A | A | E | A |
| PV022124 | 2019 | Myanmar | Yunnan | Indian Ocean | 11,237 | NSP1-E1 | D | L | T | A | A | E | A |
| PV022125 | 2019 | Myanmar | Yunnan | Indian Ocean | 11,237 | NSP1-E1 | D | L | T | A | A | E | A |
| PV022126 | 2019 | Myanmar | Yunnan | Indian Ocean | 11,237 | NSP1-E1 | D | L | T | A | A | E | A |
| PV022127 | 2019 | Myanmar | Yunnan | Indian Ocean | 11,237 | NSP1-E1 | D | L | T | A | A | E | A |
| MN871964 | 2019 | Myanmar | Taiwan | Indian Ocean | 3747 | C-E1 | D | L | T | A | A | E | A |
| MN871965 | 2019 | Myanmar | Taiwan | Indian Ocean | 3747 | C-E1 | D | L | T | A | A | E | A |
| MN871966 | 2019 | Myanmar | Taiwan | Indian Ocean | 3747 | C-E1 | D | L | T | A | A | E | A |
| MN871967 | 2019 | Myanmar | Taiwan | Indian Ocean | 3747 | C-E1 | D | L | T | A | A | E | A |
| MN871968 | 2019 | Myanmar | Taiwan | Indian Ocean | 3747 | C-E1 | D | L | T | A | A | E | A |
| MN871969 | 2019 | Myanmar | Taiwan | Indian Ocean | 3747 | C-E1 | D | L | T | A | A | E | A |
| MN871970 | 2019 | Myanmar | Taiwan | Indian Ocean | 3747 | C-E1 | D | L | T | A | A | E | A |
| MN871971 | 2019 | Thailand | Taiwan | Indian Ocean | 3747 | C-E1 | D | L | T | A | A | E | A |
| MN871972 | 2019 | Thailand | Taiwan | Indian Ocean | 3747 | C-E1 | D | L | T | A | A | E | A |
| MN871973 | 2019 | Thailand | Taiwan | Indian Ocean | 3747 | C-E1 | D | L | T | A | A | E | A |
| MN871974 | 2019 | Thailand | Taiwan | Indian Ocean | 3747 | C-E1 | D | L | T | A | A | E | A |
| MN871975 | 2019 | Malaysia | Taiwan | Indian Ocean | 3747 | C-E1 | D | L | T | A | A | E | A |
| MN432879 | 2019 | Myanmar | Henan | Indian Ocean | 1320 | E1 | - | - | - | - | A | E | A |
| PV022121 | 2020 | Myanmar | Yunnan | Indian Ocean | 11,237 | NSP1-E1 | D | L | T | A | A | E | A |
| PV022122 | 2020 | Myanmar | Yunnan | Indian Ocean | 11,237 | NSP1-E1 | D | L | T | A | A | E | A |
| PV022123 | 2020 | Cambodia | Yunnan | Indian Ocean | 11,237 | NSP1-E1 | D | L | T | A | A | E | A |
| OR785139 | 2023 | Philippines | Guangdong | Asian | 11,897 | NSP1-E1 | D | L | T | V | T | E | A |
| OR715104 | 2023 | India | Guangdong | Indian Ocean | 11,751 | NSP1-E1 | D | L | T | A | A | E | A |
| PP798381 | 2024 | Timor-Leste | Guangdong | Asian | 12,000 | NSP1-E1 | D | S | T | V | T | E | A |
| PP554682 | 2024 | Indonesia | Guangdong | Asian | 11,716 | NSP1-E1 | D | S | T | V | T | E | A |
| C_AA119977 | 2024 | unknown | Guangdong | Indian Ocean | 11,823 | NSP1-E1 | D | L | T | A | A | E | A |
| C_AA119975 | 2024 | unknown | Guangdong | Indian Ocean | 11,807 | NSP1-E1 | D | L | T | A | A | E | A |
| C_AA119979 | 2025 | unknown | Guangdong | Asian | 12,021 | NSP1-E1 | D | S | T | V | T | E | A |
| C_AA119981 | 2025 | unknown | Guangdong | Asian | 12,007 | NSP1-E1 | D | L | T | V | T | E | A |
| C_AA131420 | 2025 | unknown | Guangdong | Asian | 11,985 | NSP1-E1 | D | L | T | V | T | E | A |
| EPI_ISL_20136536 | 2025 | Indonesia | Jiangsu | Asian | 11,973 | NSP1-E1 | D | S | T | V | T | E | A |
| PV805110 | 2025 | unknown | Shanghai | Asian | 11,891 | NSP1-E1 | D | L | T | V | T | E | A |
| C_AA119978 | 2025 | unknown | Guangdong | Asian | 11,818 | NSP1-E1 | D | L | T | V | T | E | A |
| C_AA131418 | 2025 | unknown | Guangdong | Asian | 11,717 | NSP1-E1 | D | L | T | V | T | E | A |
| C_AA131421 | 2025 | unknown | Guangdong | Asian | 11,714 | NSP1-E1 | D | L | T | V | T | E | A |
| C_AA131419 | 2025 | unknown | Guangdong | Asian | 11,713 | NSP1-E1 | D | L | T | V | T | E | A |
| PX921692 | 2025 | Myanmar | Yunnan | Asian | 11,706 | NSP1-E1 | D | S | T | V | T | E | A |
| C_AA119980 | 2025 | unknown | Guangdong | Central African | 11,930 | NSP1-E1 | D | Q | T | V | A | K | V |
| C_AA131414 | 2025 | unknown | Guangdong | Central African | 11,715 | NSP1-E1 | D | Q | T | V | A | K | V |
| C_AA131428 | 2025 | unknown | Guangdong | Central African | 11,715 | NSP1-E1 | D | Q | T | V | A | K | V |
| C_AA130858 | 2025 | unknown | Ningxia | Central African | 11,281 | NSP1-E1 | D | Q | T | V | A | K | V |
| C_AA119984 | 2025 | unknown | Guangdong | Indian Ocean | 12,012 | NSP1-E1 | D | L | T | A | A | E | A |
| PX425075 | 2025 | unknown | Jiangsu | Indian Ocean | 11,812 | NSP1-E1 | D | L | T | A | A | E | A |
| C_AA119983 | 2025 | unknown | Guangdong | Indian Ocean | 11,769 | NSP1-E1 | D | L | T | A | A | E | A |
| C_AA131422 | 2025 | unknown | Guangdong | Indian Ocean | 11,715 | NSP1-E1 | D | L | T | A | A | E | A |
| C_AA131423 | 2025 | unknown | Guangdong | Indian Ocean | 11,715 | NSP1-E1 | D | L | T | A | A | E | A |
| C_AA131425 | 2025 | unknown | Guangdong | Indian Ocean | 11,715 | NSP1-E1 | D | L | T | A | A | E | A |
| C_AA131424 | 2025 | unknown | Guangdong | Indian Ocean | 11,714 | NSP1-E1 | D | L | T | A | A | E | A |
| C_AA131426 | 2025 | unknown | Guangdong | Indian Ocean | 11,714 | NSP1-E1 | D | L | T | A | A | E | A |
| C_AA131415 | 2025 | unknown | Guangdong | Indian Ocean | 11,712 | NSP1-E1 | D | L | T | A | A | E | A |
| C_AA131416 | 2025 | unknown | Guangdong | Indian Ocean | 11,711 | NSP1-E1 | D | L | T | A | A | E | A |
| C_AA131417 | 2025 | unknown | Guangdong | Indian Ocean | 11,711 | NSP1-E1 | D | L | T | A | A | E | A |
| C_AA131427 | 2025 | unknown | Guangdong | Indian Ocean | 11,711 | NSP1-E1 | D | L | T | A | A | E | A |
| C_AA119976 | 2025 | unknown | Guangdong | Indian Ocean | 11,692 | NSP1-E1 | D | L | T | A | A | E | A |
| C_AA119982 | 2025 | unknown | Guangdong | Indian Ocean | 11,638 | NSP1-E1 | D | L | T | A | A | E | A |
