# Supplementary material for: Genomic epidemiology reveals the origins and transmission dynamics of chikungunya virus in China
Source: Infect Dis Poverty. 2026 Jun 4;15:64. doi: 10.1186/s40249-026-01465-2 (PMC13234983; doi:10.1186/s40249-026-01465-2)
Supplement: Supplementary file 7 — Supplementary material 7: Table S7. Confirmed routes of cross-regional transmission for each CHIKV genotype. [file 40249_2026_1465_MOESM7_ESM.docx]

**Table S5.** GenBank and GenBase accession numbers of CHIKV sequences used in this study and amino acid variation at positively selected sites in E1 and E2 proteins (local transmission cases).

| **Accession No.** | **Collection Date** | **Province of Local Transmission (China)** | **Genotype** | **Length (nt)** | **Genomic region** | **Amino acid positions under positive selection** | | | | | | |
| --- | --- | --- | --- | --- | --- | --- | --- | --- | --- | --- | --- | --- |
|  |  |  |  |  |  | **E2** | | | | **E1** | | |
|  |  |  |  |  |  | **60** | **210** | **211** | **264** | **98** | **211** | **226** |
| JX088705 | 2010 | Guangdong | Indian Ocean | 11,811 | NSP1-E1 | D | L | T | V | A | K | V |
| PX108307 | 2010 | Guangdong | Indian Ocean | 11,770 | NSP1-E1 | D | L | T | V | A | K | V |
| HQ846356 | 2010 | Guangdong | Indian Ocean | 11,746 | NSP1-E1 | D | L | T | V | A | K | V |
| HQ846358 | 2010 | Guangdong | Indian Ocean | 11,730 | NSP1-E1 | D | L | T | V | A | K | V |
| HQ846359 | 2010 | Guangdong | Indian Ocean | 11,725 | NSP1-E1 | D | L | T | V | A | K | V |
| HQ846357 | 2010 | Guangdong | Indian Ocean | 11,720 | NSP1-E1 | D | L | T | V | A | K | V |
| JQ065888 | 2010 | Guangdong | Indian Ocean | 11,629 | NSP1-E1 | D | L | T | V | A | K | V |
| JQ065889 | 2010 | Guangdong | Indian Ocean | 11,628 | NSP1-E1 | D | L | T | V | A | K | V |
| JQ065885 | 2010 | Guangdong | Indian Ocean | 11,624 | NSP1-E1 | D | L | T | V | A | K | V |
| JQ065886 | 2010 | Guangdong | Indian Ocean | 11,624 | NSP1-E1 | D | L | T | V | A | K | V |
| JQ065887 | 2010 | Guangdong | Indian Ocean | 11,624 | NSP1-E1 | D | L | T | V | A | K | V |
| HQ659775 | 2010 | Guangdong | Indian Ocean | 355 | E1 (partial) | - | - | - | - | A | - | - |
| HQ659772 | 2010 | Guangdong | Indian Ocean | 337 | E1 (partial) | - | - | - | - | A | - | - |
| HQ659773 | 2010 | Guangdong | Indian Ocean | 336 | E1 (partial) | - | - | - | - | A | - | - |
| HQ392518 | 2010 | Guangdong | Indian Ocean | 334 | E1 (partial) | - | - | - | - | A | - | - |
| HQ659770 | 2010 | Guangdong | Indian Ocean | 334 | E1 (partial) | - | - | - | - | A | - | - |
| HQ392519 | 2010 | Guangdong | Indian Ocean | 332 | E1 (partial) | - | - | - | - | A | - | - |
| HQ392520 | 2010 | Guangdong | Indian Ocean | 332 | E1 (partial) | - | - | - | - | A | - | - |
| HQ659774 | 2010 | Guangdong | Indian Ocean | 332 | E1 (partial) | - | - | - | - | A | - | - |
| HQ392517 | 2010 | Guangdong | Indian Ocean | 326 | E1 (partial) | - | - | - | - | A | - | - |
| HQ659771 | 2010 | Guangdong | Indian Ocean | 323 | E1 (partial) | - | - | - | - | A | - | - |
| MT123009 | 2017 | Zhejiang | Indian Ocean | 11,787 | NSP1-E1 | D | L | T | A | A | E | A |
| MT123010 | 2017 | Zhejiang | Indian Ocean | 11,787 | NSP1-E1 | D | L | T | A | A | E | A |
| MT123008 | 2017 | Zhejiang | Indian Ocean | 11,787 | NSP1-E1 | D | L | T | A | A | E | A |
| OK316989 | 2019 | Yunnan | Asian | 12,018 | NSP1-E1 | D | L | T | V | T | E | A |
| OK316991 | 2019 | Yunnan | Asian | 12,018 | NSP1-E1 | D | L | T | V | T | E | A |
| OK316992 | 2019 | Yunnan | Asian | 12,018 | NSP1-E1 | D | L | T | V | T | E | A |
| OK316995 | 2019 | Yunnan | Asian | 12,018 | NSP1-E1 | D | L | T | V | T | E | A |
| OK316990 | 2019 | Yunnan | Asian | 12,017 | NSP1-E1 | D | L | T | V | T | E | A |
| OK316993 | 2019 | Yunnan | Asian | 12,017 | NSP1-E1 | D | L | T | V | T | E | A |
| OK316996 | 2019 | Yunnan | Asian | 12,017 | NSP1-E1 | D | L | T | V | T | E | A |
| OK316994 | 2019 | Yunnan | Asian | 12,016 | NSP1-E1 | D | L | T | V | T | E | A |
| MW291576 | 2019 | Yunnan | Indian Ocean | 11,812 | NSP1-E1 | D | L | T | A | A | E | A |
| MW248363 | 2019 | Yunnan | Indian Ocean | 11,812 | NSP1-E1 | D | L | T | A | A | E | A |
| PV879489 | 2019 | Yunnan | Indian Ocean | 11,812 | NSP1-E1 | D | L | T | A | A | E | A |
| PV879496 | 2019 | Yunnan | Indian Ocean | 11,807 | NSP1-E1 | D | L | T | A | A | E | A |
| PV879480 | 2019 | Yunnan | Indian Ocean | 11,801 | NSP1-E1 | D | L | T | A | A | E | A |
| MW110472 | 2019 | Yunnan | Indian Ocean | 11,789 | NSP1-E1 | D | L | T | A | A | E | A |
| MW110474 | 2019 | Yunnan | Indian Ocean | 11,789 | NSP1-E1 | D | L | T | A | A | E | A |
| MW110475 | 2019 | Yunnan | Indian Ocean | 11,789 | NSP1-E1 | D | L | T | A | A | E | A |
| MW110477 | 2019 | Yunnan | Indian Ocean | 11,789 | NSP1-E1 | D | L | T | A | A | E | A |
| PP501552 | 2019 | Yunnan | Indian Ocean | 11,789 | NSP1-E1 | D | L | T | A | A | E | A |
| MW110473 | 2019 | Yunnan | Indian Ocean | 11,789 | NSP1-E1 | D | L | T | A | A | E | A |
| MW110476 | 2019 | Yunnan | Indian Ocean | 11,789 | NSP1-E1 | D | L | T | A | A | E | A |
| PP501553 | 2019 | Yunnan | Indian Ocean | 11,781 | NSP1-E1 | D | L | T | A | A | E | A |
| PP501554 | 2019 | Yunnan | Indian Ocean | 11,781 | NSP1-E1 | D | L | T | A | A | E | A |
| MW248364 | 2019 | Yunnan | Indian Ocean | 11,779 | NSP1-E1 | D | L | T | A | A | E | A |
| PV879492 | 2019 | Yunnan | Indian Ocean | 11,744 | NSP1-E1 | D | L | T | A | A | E | A |
| PV879479 | 2019 | Yunnan | Indian Ocean | 11,716 | NSP1-E1 | D | L | T | A | A | E | A |
| PV879493 | 2019 | Yunnan | Indian Ocean | 11,667 | NSP1-E1 | D | L | T | A | A | E | A |
| PV879497 | 2019 | Yunnan | Indian Ocean | 11,368 | NSP1-E1 | D | L | T | A | A | E | A |
| PV879485 | 2019 | Yunnan | Indian Ocean | 11,133 | NSP1-E1 | D | L | T | A | A | E | A |
| PV879498 | 2019 | Yunnan | Indian Ocean | 11,107 | NSP1-E1 | D | L | T | A | A | E | A |
| PV879494 | 2019 | Yunnan | Indian Ocean | 10,933 | NSP1-E1 | D | L | T | A | A | E | A |
| PV879490 | 2019 | Yunnan | Indian Ocean | 6550 | NSP1-NSP4 | - | - | - | - | - | - | - |
| PV879488 | 2019 | Yunnan | Indian Ocean | 4078 | NSP1-NSP3 | - | - | - | - | - | - | - |
| MN871956 | 2019 | Taiwan | Indian Ocean | 3747 | C-E1 | D | L | T | A | A | E | A |
| MN871957 | 2019 | Taiwan | Indian Ocean | 3747 | C-E1 | D | L | T | A | A | E | A |
| MN871958 | 2019 | Taiwan | Indian Ocean | 3747 | C-E1 | D | L | T | A | A | E | A |
| MN871959 | 2019 | Taiwan | Indian Ocean | 3747 | C-E1 | D | L | T | A | A | E | A |
| MN871960 | 2019 | Taiwan | Indian Ocean | 3747 | C-E1 | D | L | T | A | A | E | A |
| MN871961 | 2019 | Taiwan | Indian Ocean | 3747 | C-E1 | D | L | T | A | A | E | A |
| MN871962 | 2019 | Taiwan | Indian Ocean | 3747 | C-E1 | D | L | T | A | A | E | A |
| MN871963 | 2019 | Taiwan | Indian Ocean | 3747 | C-E1 | D | L | T | A | A | E | A |
| PV879491 | 2019 | Yunnan | Indian Ocean | 3186 | C-E1 | D | L | T | A | A | E | A |
| PV879487 | 2019 | Yunnan | Indian Ocean | 3021 | E2-E1 (partial) | D | L | T | A | A | E | - |
| PV879483 | 2019 | Yunnan | Indian Ocean | 2905 | E2-E1 (partial) | G | L | T | A | A | - | - |
| PV879495 | 2019 | Yunnan | Indian Ocean | 1989 | NSP2 | - | - | - | - | - | - | - |
| PV879484 | 2019 | Yunnan | Indian Ocean | 1833 | NSP4 (partial) | - | - | - | - | - | - | - |
| MN747049 | 2019 | Yunnan | Indian Ocean | 1320 | E1 | - | - | - | - | A | E | A |
| MN747050 | 2019 | Yunnan | Indian Ocean | 1320 | E1 | - | - | - | - | A | E | A |
| MN747051 | 2019 | Yunnan | Indian Ocean | 1320 | E1 | - | - | - | - | A | E | A |
| MN747052 | 2019 | Yunnan | Indian Ocean | 1320 | E1 | - | - | - | - | A | E | A |
| MN747053 | 2019 | Yunnan | Indian Ocean | 1320 | E1 | - | - | - | - | A | E | A |
| MN747054 | 2019 | Yunnan | Indian Ocean | 1320 | E1 | - | - | - | - | A | E | A |
| MN747055 | 2019 | Yunnan | Indian Ocean | 1320 | E1 | - | - | - | - | A | E | A |
| MN747056 | 2019 | Yunnan | Indian Ocean | 1320 | E1 | - | - | - | - | A | E | A |
| MN747057 | 2019 | Yunnan | Indian Ocean | 1320 | E1 | - | - | - | - | A | E | A |
| MN747058 | 2019 | Yunnan | Indian Ocean | 1320 | E1 | - | - | - | - | A | E | A |
| MN747059 | 2019 | Yunnan | Indian Ocean | 1320 | E1 | - | - | - | - | A | E | A |
| MN747060 | 2019 | Yunnan | Indian Ocean | 1320 | E1 | - | - | - | - | A | E | A |
| MN747061 | 2019 | Yunnan | Indian Ocean | 1320 | E1 | - | - | - | - | A | E | A |
| MN747062 | 2019 | Yunnan | Indian Ocean | 1320 | E1 | - | - | - | - | A | E | A |
| MN747063 | 2019 | Yunnan | Indian Ocean | 1320 | E1 | - | - | - | - | A | E | A |
| MN747064 | 2019 | Yunnan | Indian Ocean | 1320 | E1 | - | - | - | - | A | E | A |
| MN747065 | 2019 | Yunnan | Indian Ocean | 1320 | E1 | - | - | - | - | A | E | A |
| MN747066 | 2019 | Yunnan | Indian Ocean | 1320 | E1 | - | - | - | - | A | E | A |
| MN747067 | 2019 | Yunnan | Indian Ocean | 1320 | E1 | - | - | - | - | A | E | A |
| MN747068 | 2019 | Yunnan | Indian Ocean | 1320 | E1 | - | - | - | - | A | E | A |
| MN747069 | 2019 | Yunnan | Indian Ocean | 1320 | E1 | - | - | - | - | A | E | A |
| MN747070 | 2019 | Yunnan | Indian Ocean | 1320 | E1 | - | - | - | - | A | E | A |
| MN747071 | 2019 | Yunnan | Indian Ocean | 1320 | E1 | - | - | - | - | A | E | A |
| MN747072 | 2019 | Yunnan | Indian Ocean | 1320 | E1 | - | - | - | - | A | E | A |
| MN747073 | 2019 | Yunnan | Indian Ocean | 1320 | E1 | - | - | - | - | A | E | A |
| MN747074 | 2019 | Yunnan | Indian Ocean | 1320 | E1 | - | - | - | - | A | E | A |
| MN747075 | 2019 | Yunnan | Indian Ocean | 1320 | E1 | - | - | - | - | A | E | A |
| MN747076 | 2019 | Yunnan | Indian Ocean | 1320 | E1 | - | - | - | - | A | E | A |
| MN747077 | 2019 | Yunnan | Indian Ocean | 1320 | E1 | - | - | - | - | A | E | A |
| MN747078 | 2019 | Yunnan | Indian Ocean | 1320 | E1 | - | - | - | - | A | E | A |
| MN747079 | 2019 | Yunnan | Indian Ocean | 1320 | E1 | - | - | - | - | A | E | A |
| MN747080 | 2019 | Yunnan | Indian Ocean | 1320 | E1 | - | - | - | - | A | E | A |
| MN747081 | 2019 | Yunnan | Indian Ocean | 1320 | E1 | - | - | - | - | A | E | A |
| MN747082 | 2019 | Yunnan | Indian Ocean | 1320 | E1 | - | - | - | - | A | E | A |
| MN747083 | 2019 | Yunnan | Indian Ocean | 1320 | E1 | - | - | - | - | A | E | A |
| MN747084 | 2019 | Yunnan | Indian Ocean | 1320 | E1 | - | - | - | - | A | E | A |
| MN747085 | 2019 | Yunnan | Indian Ocean | 1320 | E1 | - | - | - | - | A | E | A |
| PV879481 | 2019 | Yunnan | Indian Ocean | 1106 | E2-E1 (partial) | - | - | - | - | A | - | - |
| PV879482 | 2019 | Yunnan | Indian Ocean | 886 | NSP4 (partial) | - | - | - | - | - | - | - |
| PV879486 | 2019 | Yunnan | Indian Ocean | 300 | C (partial) | - | - | - | - | - | - | - |
| PX216392 | 2025 | Guangdong | Central African | 11,713 | NSP1-E1 | D | Q | T | V | A | K | V |
| PX216393 | 2025 | Guangdong | Central African | 11,713 | NSP1-E1 | D | Q | T | V | A | K | V |
| C_AA120363 | 2025 | Guangdong | Central African | 11,713 | NSP1-E1 | D | Q | T | V | A | K | V |
| C_AA120362 | 2025 | Guangdong | Central African | 11,713 | NSP1-E1 | D | Q | T | V | A | K | V |
| PX216394 | 2025 | Guangdong | Central African | 11,701 | NSP1-E1 | D | Q | T | V | A | K | V |
| PX216391 | 2025 | Guangdong | Central African | 11,701 | NSP1-E1 | D | Q | T | V | A | K | V |
| C_AA119378 | 2025 | Guangdong | Central African | 11,422 | NSP1-E1 | D | Q | T | V | A | K | V |
| C_AA119405 | 2025 | Guangdong | Central African | 11,422 | NSP1-E1 | D | Q | T | V | A | K | V |
| C_AA119407 | 2025 | Guangdong | Central African | 11,422 | NSP1-E1 | D | Q | T | V | A | K | V |
| C_AA119412 | 2025 | Guangdong | Central African | 11,422 | NSP1-E1 | D | Q | T | V | A | K | V |
| C_AA119423 | 2025 | Guangdong | Central African | 11,422 | NSP1-E1 | D | Q | T | V | A | K | V |
| C_AA119424 | 2025 | Guangdong | Central African | 11,422 | NSP1-E1 | D | Q | T | V | A | K | V |
| C_AA119435 | 2025 | Guangdong | Central African | 11,422 | NSP1-E1 | D | Q | T | V | A | K | V |
| C_AA119436 | 2025 | Guangdong | Central African | 11,422 | NSP1-E1 | D | Q | T | V | A | K | V |
| C_AA119450 | 2025 | Guangdong | Central African | 11,422 | NSP1-E1 | D | Q | T | V | A | K | V |
| C_AA119452 | 2025 | Guangdong | Central African | 11,422 | NSP1-E1 | D | Q | T | V | A | K | V |
| C_AA119460 | 2025 | Guangdong | Central African | 11,422 | NSP1-E1 | D | Q | T | V | A | K | V |
| C_AA119375 | 2025 | Guangdong | Central African | 11,422 | NSP1-E1 | D | Q | T | V | A | K | V |
| C_AA119385 | 2025 | Guangdong | Central African | 11,422 | NSP1-E1 | D | Q | T | V | A | K | V |
| C_AA119386 | 2025 | Guangdong | Central African | 11,422 | NSP1-E1 | D | Q | T | V | A | K | V |
| C_AA119390 | 2025 | Guangdong | Central African | 11,422 | NSP1-E1 | D | Q | T | V | A | K | V |
| C_AA119394 | 2025 | Guangdong | Central African | 11,422 | NSP1-E1 | D | Q | T | V | A | K | V |
| C_AA119404 | 2025 | Guangdong | Central African | 11,422 | NSP1-E1 | D | Q | T | V | A | K | V |
| C_AA119406 | 2025 | Guangdong | Central African | 11,422 | NSP1-E1 | D | Q | T | V | A | K | V |
| C_AA119411 | 2025 | Guangdong | Central African | 11,422 | NSP1-E1 | D | Q | T | V | A | K | V |
| C_AA119413 | 2025 | Guangdong | Central African | 11,422 | NSP1-E1 | D | Q | T | V | A | K | V |
| C_AA119421 | 2025 | Guangdong | Central African | 11,422 | NSP1-E1 | D | Q | T | V | A | K | V |
| C_AA119428 | 2025 | Guangdong | Central African | 11,422 | NSP1-E1 | D | Q | T | V | A | K | V |
| C_AA119376 | 2025 | Guangdong | Central African | 11,422 | NSP1-E1 | D | Q | T | V | A | K | V |
| C_AA119377 | 2025 | Guangdong | Central African | 11,422 | NSP1-E1 | D | Q | T | V | A | K | V |
| C_AA119389 | 2025 | Guangdong | Central African | 11,422 | NSP1-E1 | D | Q | T | V | A | K | V |
| C_AA119391 | 2025 | Guangdong | Central African | 11,422 | NSP1-E1 | D | Q | T | V | A | K | V |
| C_AA119402 | 2025 | Guangdong | Central African | 11,422 | NSP1-E1 | D | Q | T | V | A | K | V |
| C_AA119408 | 2025 | Guangdong | Central African | 11,422 | NSP1-E1 | D | Q | T | V | A | K | V |
| C_AA119414 | 2025 | Guangdong | Central African | 11,422 | NSP1-E1 | D | Q | T | V | A | K | V |
| C_AA119417 | 2025 | Guangdong | Central African | 11,422 | NSP1-E1 | D | Q | T | V | A | K | V |
| C_AA119422 | 2025 | Guangdong | Central African | 11,422 | NSP1-E1 | D | Q | T | V | A | K | V |
| C_AA119431 | 2025 | Guangdong | Central African | 11,422 | NSP1-E1 | D | Q | T | V | A | K | V |
| C_AA119444 | 2025 | Guangdong | Central African | 11,422 | NSP1-E1 | D | Q | T | V | A | K | V |
| C_AA119449 | 2025 | Guangdong | Central African | 11,422 | NSP1-E1 | D | Q | T | V | A | K | V |
| C_AA119456 | 2025 | Guangdong | Central African | 11,422 | NSP1-E1 | D | Q | T | V | A | K | V |
| C_AA119457 | 2025 | Guangdong | Central African | 11,422 | NSP1-E1 | D | Q | T | V | A | K | V |
| C_AA119372 | 2025 | Guangdong | Central African | 11,422 | NSP1-E1 | D | Q | T | V | A | K | V |
| C_AA119373 | 2025 | Guangdong | Central African | 11,422 | NSP1-E1 | D | Q | T | V | A | K | V |
| C_AA119398 | 2025 | Guangdong | Central African | 11,422 | NSP1-E1 | D | Q | T | V | A | K | V |
| C_AA119399 | 2025 | Guangdong | Central African | 11,422 | NSP1-E1 | D | Q | T | V | A | K | V |
| C_AA119410 | 2025 | Guangdong | Central African | 11,422 | NSP1-E1 | D | Q | T | V | A | K | V |
| C_AA119441 | 2025 | Guangdong | Central African | 11,422 | NSP1-E1 | D | Q | T | V | A | K | V |
| C_AA119448 | 2025 | Guangdong | Central African | 11,422 | NSP1-E1 | D | Q | T | V | A | K | V |
| C_AA119459 | 2025 | Guangdong | Central African | 11,422 | NSP1-E1 | D | Q | T | V | A | K | V |
| C_AA119382 | 2025 | Guangdong | Central African | 11,422 | NSP1-E1 | D | Q | T | V | A | K | V |
| C_AA119383 | 2025 | Guangdong | Central African | 11,422 | NSP1-E1 | D | Q | T | V | A | K | V |
| C_AA119392 | 2025 | Guangdong | Central African | 11,422 | NSP1-E1 | D | Q | T | V | A | K | V |
| C_AA119393 | 2025 | Guangdong | Central African | 11,422 | NSP1-E1 | D | Q | T | V | A | K | V |
| C_AA119401 | 2025 | Guangdong | Central African | 11,422 | NSP1-E1 | D | Q | T | V | A | K | V |
| C_AA119409 | 2025 | Guangdong | Central African | 11,422 | NSP1-E1 | D | Q | T | V | A | K | V |
| C_AA119426 | 2025 | Guangdong | Central African | 11,422 | NSP1-E1 | D | Q | T | V | A | K | V |
| C_AA119430 | 2025 | Guangdong | Central African | 11,422 | NSP1-E1 | D | Q | T | V | A | K | V |
| C_AA119433 | 2025 | Guangdong | Central African | 11,422 | NSP1-E1 | D | Q | T | V | A | K | V |
| C_AA119438 | 2025 | Guangdong | Central African | 11,422 | NSP1-E1 | D | Q | T | V | A | K | V |
| C_AA119455 | 2025 | Guangdong | Central African | 11,422 | NSP1-E1 | D | Q | T | V | A | K | V |
| C_AA119461 | 2025 | Guangdong | Central African | 11,422 | NSP1-E1 | D | Q | T | V | A | K | V |
| C_AA119379 | 2025 | Guangdong | Central African | 11,422 | NSP1-E1 | D | Q | T | V | A | K | V |
| C_AA119416 | 2025 | Guangdong | Central African | 11,422 | NSP1-E1 | D | Q | T | V | A | K | V |
| C_AA119451 | 2025 | Guangdong | Central African | 11,422 | NSP1-E1 | D | Q | T | V | A | K | V |
| C_AA119454 | 2025 | Guangdong | Central African | 11,422 | NSP1-E1 | D | Q | T | V | A | K | V |
| C_AA119371 | 2025 | Guangdong | Central African | 11,422 | NSP1-E1 | D | Q | T | V | A | K | V |
| C_AA119387 | 2025 | Guangdong | Central African | 11,422 | NSP1-E1 | D | Q | T | V | A | K | V |
| C_AA119397 | 2025 | Guangdong | Central African | 11,422 | NSP1-E1 | D | Q | T | V | A | K | V |
| C_AA119400 | 2025 | Guangdong | Central African | 11,422 | NSP1-E1 | D | Q | T | V | A | K | V |
| C_AA119446 | 2025 | Guangdong | Central African | 11,422 | NSP1-E1 | D | Q | T | V | A | K | V |
| C_AA119447 | 2025 | Guangdong | Central African | 11,422 | NSP1-E1 | D | Q | T | V | A | K | V |
| C_AA119453 | 2025 | Guangdong | Central African | 11,422 | NSP1-E1 | D | Q | T | V | A | K | V |
| C_AA119396 | 2025 | Guangdong | Central African | 11,422 | NSP1-E1 | D | Q | T | V | A | K | V |
| C_AA119415 | 2025 | Guangdong | Central African | 11,422 | NSP1-E1 | D | Q | T | V | A | K | V |
| C_AA119419 | 2025 | Guangdong | Central African | 11,422 | NSP1-E1 | D | Q | T | V | A | K | V |
| C_AA119427 | 2025 | Guangdong | Central African | 11,422 | NSP1-E1 | D | Q | T | V | A | K | V |
| C_AA119432 | 2025 | Guangdong | Central African | 11,422 | NSP1-E1 | D | Q | T | V | A | K | V |
| C_AA119439 | 2025 | Guangdong | Central African | 11,422 | NSP1-E1 | D | Q | T | V | A | K | V |
| C_AA119442 | 2025 | Guangdong | Central African | 11,422 | NSP1-E1 | D | Q | T | V | A | K | V |
| C_AA119374 | 2025 | Guangdong | Central African | 11,422 | NSP1-E1 | D | Q | T | V | A | K | V |
| C_AA119388 | 2025 | Guangdong | Central African | 11,422 | NSP1-E1 | D | Q | T | V | A | K | V |
| C_AA119395 | 2025 | Guangdong | Central African | 11,422 | NSP1-E1 | D | Q | T | V | A | K | V |
| C_AA119403 | 2025 | Guangdong | Central African | 11,422 | NSP1-E1 | D | Q | T | V | A | K | V |
| C_AA119418 | 2025 | Guangdong | Central African | 11,422 | NSP1-E1 | D | Q | T | V | A | K | V |
| C_AA119420 | 2025 | Guangdong | Central African | 11,422 | NSP1-E1 | D | Q | T | V | A | K | V |
| C_AA119429 | 2025 | Guangdong | Central African | 11,422 | NSP1-E1 | D | Q | T | V | A | K | V |
| C_AA119440 | 2025 | Guangdong | Central African | 11,422 | NSP1-E1 | D | Q | T | V | A | K | V |
| C_AA119445 | 2025 | Guangdong | Central African | 11,422 | NSP1-E1 | D | Q | T | V | A | K | V |
| C_AA119458 | 2025 | Guangdong | Central African | 11,422 | NSP1-E1 | D | Q | T | V | A | K | V |
| C_AA119380 | 2025 | Guangdong | Central African | 11,422 | NSP1-E1 | D | Q | T | V | A | K | V |
| C_AA119381 | 2025 | Guangdong | Central African | 11,422 | NSP1-E1 | D | Q | T | V | A | K | V |
| C_AA119384 | 2025 | Guangdong | Central African | 11,422 | NSP1-E1 | D | Q | T | V | A | K | V |
| C_AA119425 | 2025 | Guangdong | Central African | 11,422 | NSP1-E1 | D | Q | T | V | A | K | V |
| C_AA119434 | 2025 | Guangdong | Central African | 11,422 | NSP1-E1 | D | Q | T | V | A | K | V |
| C_AA119437 | 2025 | Guangdong | Central African | 11,422 | NSP1-E1 | D | Q | T | V | A | K | V |
| C_AA119443 | 2025 | Guangdong | Central African | 11,422 | NSP1-E1 | D | Q | T | V | A | K | V |
| C_AA119462 | 2025 | Guangdong | Central African | 11,422 | NSP1-E1 | D | Q | T | V | A | K | V |
