# Supplementary material for: Genomic epidemiology reveals the origins and transmission dynamics of chikungunya virus in China
Source: Infect Dis Poverty. 2026 Jun 4;15:64. doi: 10.1186/s40249-026-01465-2 (PMC13234983; doi:10.1186/s40249-026-01465-2)
Supplement: Supplementary file 8 — Supplementary material 8: Fig S1. Workflow for data collection, sequence processing, and phylogenetic/phylogeographic analyses of CHIKV in China. [file 40249_2026_1465_MOESM8_ESM.docx]

**Table S6.** Annual number of reported CHIKV cases and available sequences in China, 1987–2025.

| **Year** | **Reported CHIKV Cases** | **Number of Sequences** |
| --- | --- | --- |
| 1987 | 1 | 0 |
| 2006 | 4 | 1 |
| 2007 | 3 | 0 |
| 2008 | 18 | 4 |
| 2009 | 10 | 3 |
| **2010** | **327** | **26** |
| 2011 | 4 | 0 |
| 2012 | 10 | 3 |
| 2013 | 33 | 0 |
| 2014 | 10 | 0 |
| 2015 | 4 | 0 |
| 2016 | 20 | 2 |
| 2017 | 45 | 8 |
| 2018 | 16 | 1 |
| **2019** | **430** | **131** |
| 2020 | 5 | 3 |
| 2023 | 3 | 2 |
| 2024 | 6 | 4 |
| **2025** | **25,415** | **126** |
| Total | 26,364 | 314 |
