# Supplementary material for: Genomic epidemiology reveals the origins and transmission dynamics of chikungunya virus in China
Source: Infect Dis Poverty. 2026 Jun 4;15:64. doi: 10.1186/s40249-026-01465-2 (PMC13234983; doi:10.1186/s40249-026-01465-2)
Supplement: Supplementary file 9 — Supplementary material 9: Fig S2. Maximum likelihood phylogenetic analysis reveals indigenous and imported chikungunya virus lineages/branches in China. [file 40249_2026_1465_MOESM9_ESM.docx]

**Table S7** Confirmed routes of cross-regional transmission for each CHIKV genotype.

| **Genotype** | **From** | **To** | **BF*** | **PP** |
| --- | --- | --- | --- | --- |
| ECSA | **South Asia** | **Southeast Asia** | **223,684.29** | **1** |
|  | **Southeast Asia** | **Guangdong** | **55,914.86** | **0.999851852** |
|  | Yunnan | Southeast Asia | 3056.00 | 0.997296296 |
|  | South Asia | Europe | 2623.39 | 0.996851852 |
|  | South Asia | Africa | 1578.19 | 0.994777778 |
|  | **Southeast Asia** | **Yunnan** | **1503.15** | **0.994518519** |
|  | South America | North America | 739.8507831 | 0.988925926 |
|  | **Africa** | **Guangdong** | **677.8886245** | **0.987925926** |
|  | South America | Europe | 280.350667 | 0.971296296 |
|  | **Africa** | **South Asia** | **166.3384875** | **0.952555556** |
|  | Southeast Asia | Oceania | 164.7178527 | 0.952111111 |
|  | South Asia | West Asia | 94.84675071 | 0.919666667 |
|  | South Asia | North America | 43.31660601 | 0.839444444 |
|  | Africa | West Asia | 38.76863898 | 0.823925926 |
|  | Africa | South America | 5.52753649 | 0.400185185 |
|  | North America | South America | 3.349383995 | 0.287888889 |
|  | West Asia | South America | 3.313792201 | 0.285703704 |
| Asian | North America | South America | 76,846.61 | 1 |
|  | Southeast Asia | Oceania | 501.3283784 | 0.991555556 |
|  | Southeast Asia | North America | 286.8323117 | 0.985333333 |
|  | North America | Oceania | 30.91863712 | 0.878666667 |
|  | **Southeast Asia** | **Yunnan** | **14.06762843** | **0.767166667** |
|  | South Asia | Southeast Asia | 5.132375773 | 0.545888889 |

* This shows that only transmission relationships with a BF of 3 or more were chosen.
