# Supplementary material for: Genomic epidemiology reveals the origins and transmission dynamics of chikungunya virus in China
Source: Infect Dis Poverty. 2026 Jun 4;15:64. doi: 10.1186/s40249-026-01465-2 (PMC13234983; doi:10.1186/s40249-026-01465-2)

**Fig. S1** **Workflow for data collection, sequence processing, and phylogenetic/phylogeographic analyses of CHIKV in China.**


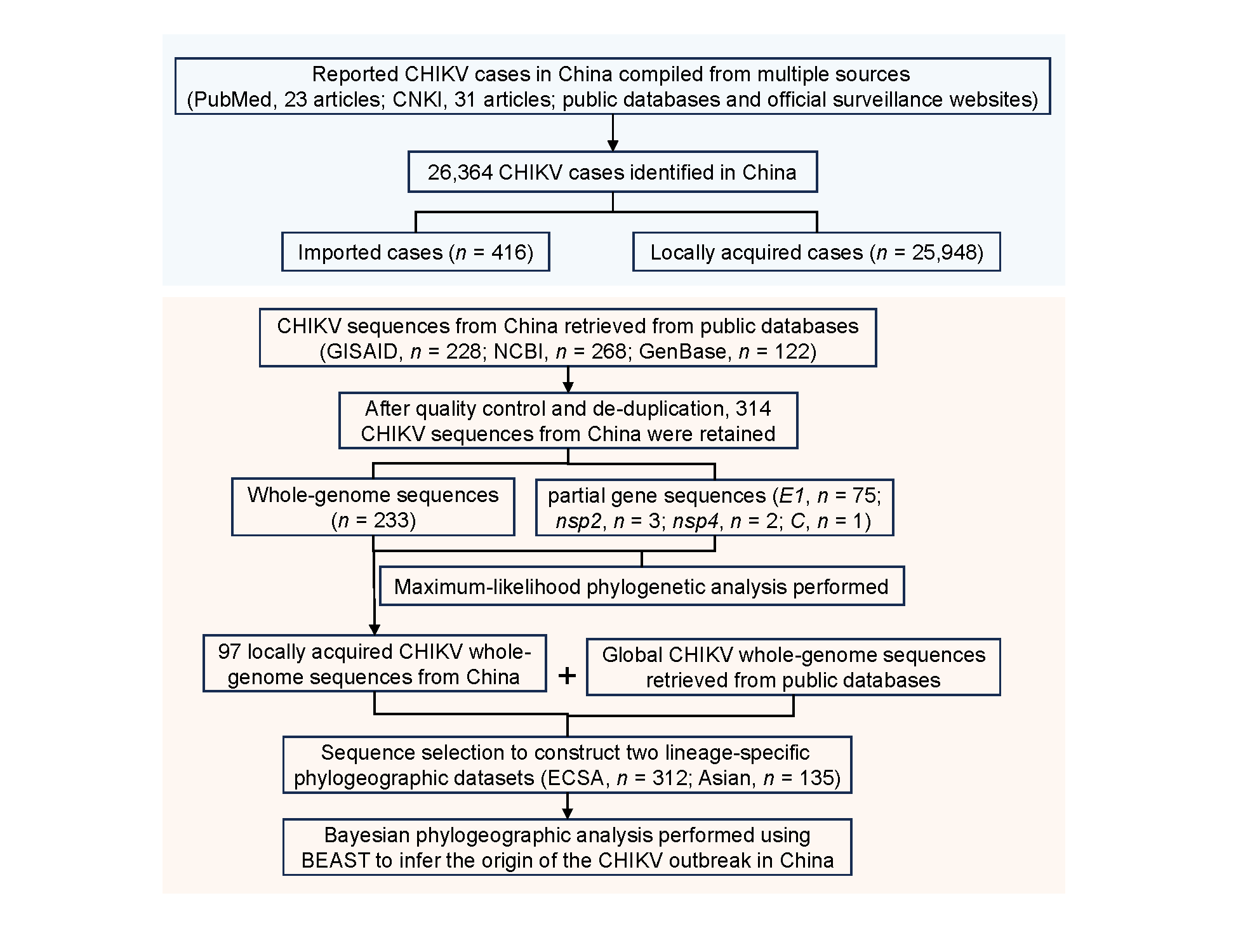

Supplement: Supplementary file 10 — Supplementary material 10: Fig S3. The Bayesian discrete geographic tree reveals the global transmission pathways of the ECSA lineage of chikungunya virus. [file 40249_2026_1465_MOESM10_ESM.docx]
