# Supplementary material for: Genomic epidemiology reveals the origins and transmission dynamics of chikungunya virus in China
Source: Infect Dis Poverty. 2026 Jun 4;15:64. doi: 10.1186/s40249-026-01465-2 (PMC13234983; doi:10.1186/s40249-026-01465-2)

**Fig. S2**. **Maximum-likelihood phylogenetic analysis reveals indigenous and imported chikungunya virus lineages/branches in China.** Phylogenetic trees are annotated with red solid circles representing local cases and green solid circles representing imported cases. (A) nt 133-11,313, (B) nt 10,259-11,293, (C) nt 1682-3670, (D) nt 10,237-10,568, (E) nt 6161-7046, (F) nt 7584-7883. Nucleotide positions are based on the L2006 strain (GenBank accession number DQ443544.2).


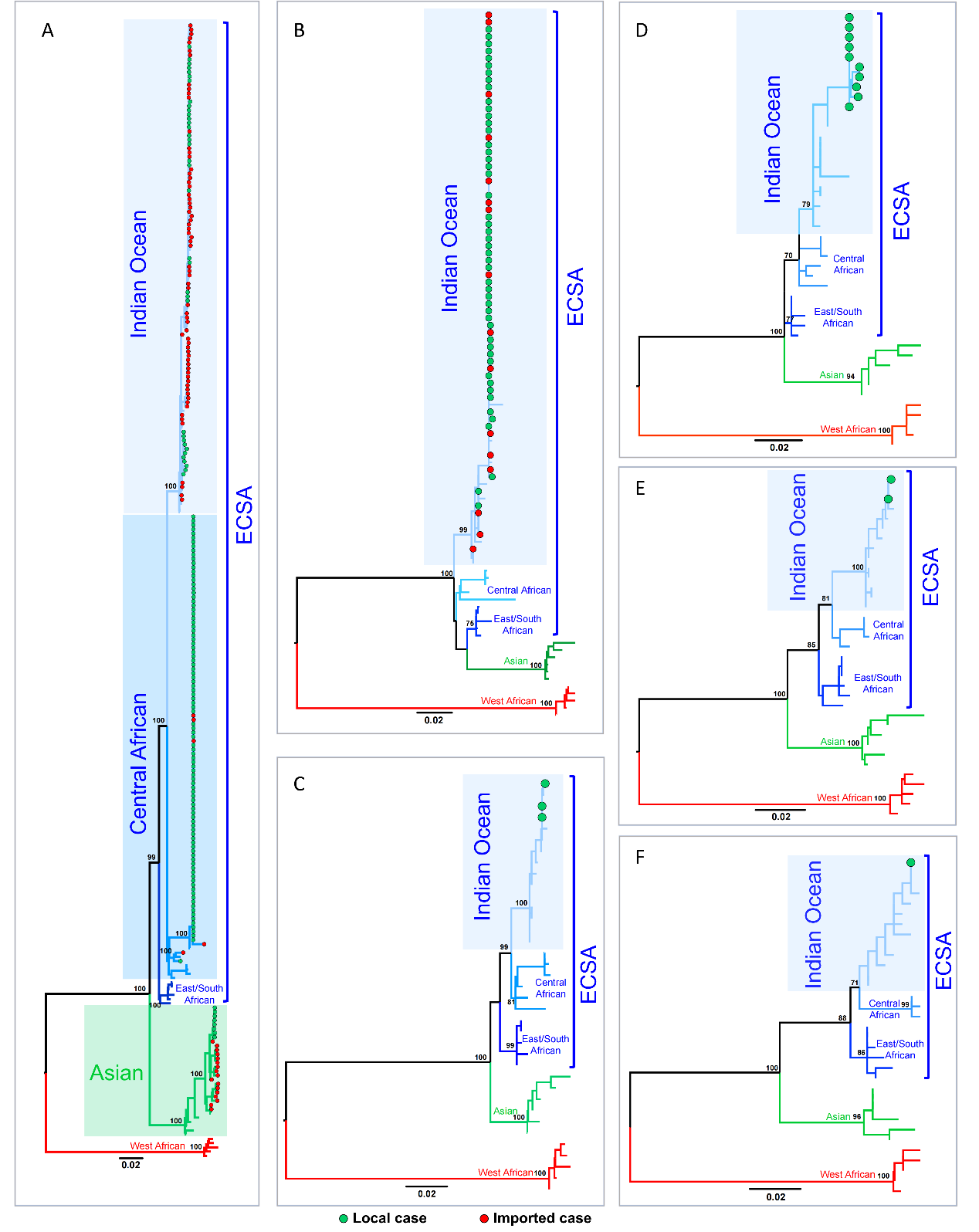

Supplement: Supplementary file 11 — Supplementary material 11: Fig S4. The Bayesian discrete geographic tree reveals the global transmission pathways of the Asian lineage of chikungunya virus. [file 40249_2026_1465_MOESM11_ESM.docx]
